# 3D Whole‐Heart Joint T_1_/T_1ρ_ Mapping and Water‐Fat Imaging on a Clinical 0.55‐T Low‐Field Scanner

**DOI:** 10.1002/nbm.70195

**Published:** 2025-12-22

**Authors:** Michael G. Crabb, Karl P. Kunze, Carlos Castillo‐Passi, Dongyue Si, Simon J. Littlewood, Claudia Prieto, René M. Botnar

**Affiliations:** ^1^ School of Biomedical Engineering and Imaging Sciences King's College London London UK; ^2^ MR Research Collaborations Siemens Healthcare Limited Camberley UK; ^3^ Institute for Biological and Medical Engineering Pontificia Universidad Católica de Chile Santiago Chile; ^4^ Millennium Institute for Intelligent Healthcare Engineering Santiago Chile; ^5^ School of Engineering Pontificia Universidad Católica de Chile Santiago Chile; ^6^ Institute for Advanced Study Technical University of Munich Germany

**Keywords:** cardiac MRI, low‐field, myocardial tissue characterisation, 3D multiparametric MRI, $T_{1}$ mapping, $T_{1\rho }$ mapping

## Abstract

Myocardial maps are conventionally acquired in 2D breath‐hold single‐parameter scans that are slow and have limited heart coverage. To overcome limitations associated with 2D breath‐hold mapping sequences, we develop a novel free‐breathing 3D joint $T_{1}$/ $T_{1\rho }$ mapping sequence with Dixon encoding to provide co‐registered 3D $T_{1}$ and $T_{1\rho }$ maps and water‐fat volumes with isotropic spatial resolution in a single scan for comprehensive contrast‐agent free myocardial tissue characterization and visualisation of the whole‐heart anatomy on a clinical 0.55‐T MR scanner. The proposed sequence acquires four interleaved 3D volumes with preparation modules to provide $T_{1}$ and $T_{1\rho }$ encoding, with data acquired with a two‐echo Dixon readout and 2D image navigators to enable 100% respiratory scan efficiency. Images were reconstructed with nonrigid respiratory motion‐corrected iterative SENSE with multi‐dimensional low‐rank patch‐based denoising, and maps generated by matching with simulated dictionaries. The proposed sequence was tested in phantoms, 11 healthy subjects and 1 patient, and compared with conventional techniques. For phantoms, the proposed 3D $T_{1}$ and $T_{1\rho }$ measurements showed good correlation with 2D spin‐echo reference measurements. For healthy subjects, septal myocardial tissue mapping values were $T_{1}=743\pm 19\;\text{ms}$ and $T_{1\rho }=46.9\pm 2.7\;\text{ms}$ for the proposed sequence, against $T_{1}=681\pm 23\;\text{ms}$ and $T_{1\rho }=57.9\pm 3.6\;\text{ms}$ for 2D modified Look‐Locker inversion recovery and 2D $T_{1\rho }$ respectively. Promising results were obtained when the proposed mapping was compared to 2D late‐gadolinium enhancement imaging in a patient. The proposed approach enables simultaneous 3D whole‐heart joint $T_{1}$/$T_{1\rho }$ mapping and water‐fat imaging at 0.55 T in a single scan of ≈11 min, demonstrating good agreement with conventional techniques in phantoms and healthy subjects, and promising results in a patient.

## Graphical abstract

A 3D joint T1/T1ρ mapping sequence was proposed at 0.55 T. Four interleaved T1 and T1ρ encoded 3D volumes are acquired; images are reconstructed with nonrigid respiratory motion‐correction, low‐rank patch‐based denoising (HD‐PROST) and water‐fat separation; and T1/T1ρ maps are estimated through dictionary matching. The proposed sequence provides 3D whole‐heart coregistered water/fat volumes and T1/T1ρ maps in a single 11‐min free‐breathing scan, and it demonstrated good agreement with conventional techniques in phantoms and healthy subjects and promising results in a patient.

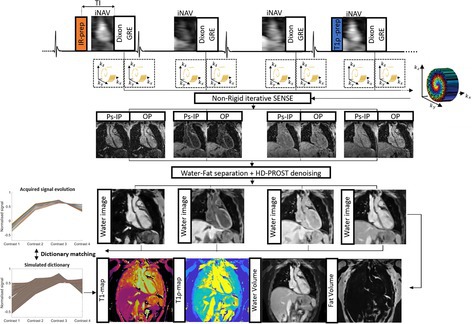

**Abbreviations**
AHA: American heart association
BW: bandwidth
CAIPIRINHA: controlled aliasing in parallel imaging results in higher acceleration
CE: contrast enhanced
CV: coefficient of variation
ECG: electrocardiogram
FA: flip angle
FOV: field of view
GRAPPA: generalised autocalibrating partially parallel acquisitions
GRE: gradient recalled echo
HD‐PROST: high‐dimensionality undersampled patch‐based reconstruction
HB: heart‐beat
HR: heart‐rate
iNAV: image navigator
IR: inversion recovery
LGE: late gadolinium enhancement
MOLLI: modified Look‐Locker inversion recovery
MRF: magnetic resonance fingerprinting
OP: out‐of‐phase
PSIR: phase sensitive inversion recovery
Ps‐IP: pseudo‐in‐phase
ROI: region of interest
SAR: specific absorption rate
SAx: short‐axis
SD: standard deviation
SE: spin‐echo
SLA: spin‐lock amplitude
SNR: signal‐to‐noise ratio
SSD: sum of squared differences
TE: echo time
TI: inversion time
TR: repetition time
TSL: spin‐lock duration
VD‐CASPR: variable density Cartesian acquisition with spiral profile order
VIBE: volumetric interpolated breath‐hold examination

## 1 Introduction

In patients with suspected myocardial infarction, late‐gadolinium enhancement (LGE) images are often acquired in concert with $T_{1}$ maps acquired pre‐gadolinium and post‐gadolinium contrast injection to detect focal and diffuse scarring [1]. In patients with a contraindication to gadolinium‐based contrast agents, such as renal dysfunction or contrast agent allergy, a noncontrast enhanced (non‐CE) approach would be of great value. Native $T_{1}$ mapping has shown promising results in noninvasive detection of a broad range of pathologies including fibrosis, oedema, fat and iron deposition [1, 2, 3, 4, 5] but is nonspecific. Native $T_{1\rho }$ mapping has recently shown potential for non‐CE imaging of fibrosis and chronic myocardial infarction [6, 7, 8, 9]$.T_{1\rho }$ uses a spin‐lock RF pulse with much lower frequency compared with the Larmor frequency and is sensitive to slow molecular motion processes such as collagen in fibrosis, but $T_{1}$ is indirectly sensitive to fibrosis through the effects of macromolecules on water [9]. Thus, an advantage of $T_{1\rho }$ mapping is that it can be used to more directly detect the presence of fibrotic tissue and has been shown to have improved specificity and sensitivity in identifying diffuse fibrosis within LGE grey area compared to native $T_{1}$ mapping [10]. The two techniques are therefore complementary in a comprehensive assessment of myocardial tissue characterisation.

Quantitative myocardial $T_{1}$ mapping techniques are typically performed under breath‐hold via the acquisition of several single‐shot images (ECG‐triggered), followed by pixelwise exponential‐type model fitting to obtain 2D maps [11, 12, 13]. $T_{1\rho }$ is less common clinically, but 2D breath‐hold sequences have also been proposed [14, 15]. 3D whole‐heart free‐breathing mapping techniques have been proposed to remove the requirement of breath‐holds, increase spatial resolution and increase heart coverage compared with 2D breath‐hold sequences. Recent examples of these developments include respiratory‐gated 3D $T_{1}$ [16] and 3D $T_{1\rho }$ [17] mapping; however, these approaches can result in long and unpredictable scan times due to respiratory gating. More recently, 3D T1 [18] and 3D T1ρ [19] mappings that compensate for respiratory motion using 2D image navigators (iNAVs) that achieve 100% respiratory scan efficiency [20] have been proposed. However, maps are acquired sequentially which can result in mis‐registration artifacts and long scan times. To address these shortcomings, 3D free‐breathing joint $T_{1}$/$T_{2}$ mapping sequences, where $T_{1}$ and $T_{2}$ are quantified in a single free‐breathing scan, have been proposed at 1.5T and 3T [21, 22]. A 3D joint $T_{1}$/$T_{1\rho }$ mapping technique has also recently been proposed at 3T [23].

However, there is very limited experience with these approaches at lower‐field strengths, which may provide more affordable access to cardiac MRI in the future [24]. Additionally, a wider bore makes low‐field scans more acceptable for bariatric and claustrophobic patients [25]. Cardiac applications have recently been implemented for a comprehensive cardiac magnetic resonance (CMR) exam on a novel 0.55‐T system [26]. At 0.55 T, there are few multiparametric MRI studies published, although feasibility of 3D whole‐heart joint $T_{1}$/$T_{2}$ mapping [27], brain $T_{1}$/$T_{2}$ magnetic resonance fingerprinting (MRF) [28], 3D liver simultaneous $T_{1}$/$T_{2}$/fat‐fraction mapping [29], liver $T_{1}$/$T_{2}$ MRF [30] and placenta combined $T_{2}^{*}$/diffusion MRI [31] have recently been demonstrated.

Despite low‐field having lower signal‐to‐noise ratio (SNR) [32], there are several physics advantages that make low‐field MRI a potentially attractive alternative for 3D joint $T_{1}$/$T_{1\rho }$ mapping including [33, 34]: (i) shorter $T_{1}$ relaxation times (for improved $T_{1}$ sensitivity, as demonstrated in Section 3.1), (ii) potentially improved $B_{0}$ and $B_{1}$ field homogeneity (to reduce artifacts in reconstructed images, and potentially improve the accuracy and precision of MR relaxation parameter estimates [35]), and (iii) reduced specific absorption rate (SAR). The main challenges that need to be addressed for 3D whole‐heart joint $T_{1}$/$T_{1\rho }$ mapping at low‐field are the lower SNR, as well as fat‐suppression. For fat suppression, the shorter $T_{1}$ of fat and the reduction in chemical shift (in Hz) between the water/fat resonance peaks makes fat suppression techniques based on spectrally selective RF pulses challenging. However, feasibility of fat‐water separation with a two echo Dixon readout has been demonstrated for body composition profiling at 0.55 T [36].

The aim of this work was to investigate the feasibility of a novel free‐breathing, 3D joint $T_{1}$/ $T_{1\rho }$ mapping sequence with Dixon encoding to provide coregistered 3D $T_{1}$ and $T_{1\rho }$ maps and water‐fat volumes with isotropic spatial resolution in a single scan for comprehensive contrast‐agent free myocardial tissue characterization and visualisation of the whole‐heart anatomy on a clinical 0.55‐T MR scanner. Patch‐based, multidimensional low‐rank denoising [37] is deployed and Dixon water‐fat separation to address the challenges of lower SNR and fat suppression respectively at low‐field. A complementary coregistered $T_{1\rho }$‐prepared bright‐blood water volume is obtained to enable whole‐heart anatomy visualisation.

## 2 Methods

### 2.1 Pulse Sequence

**FIGURE 1:**
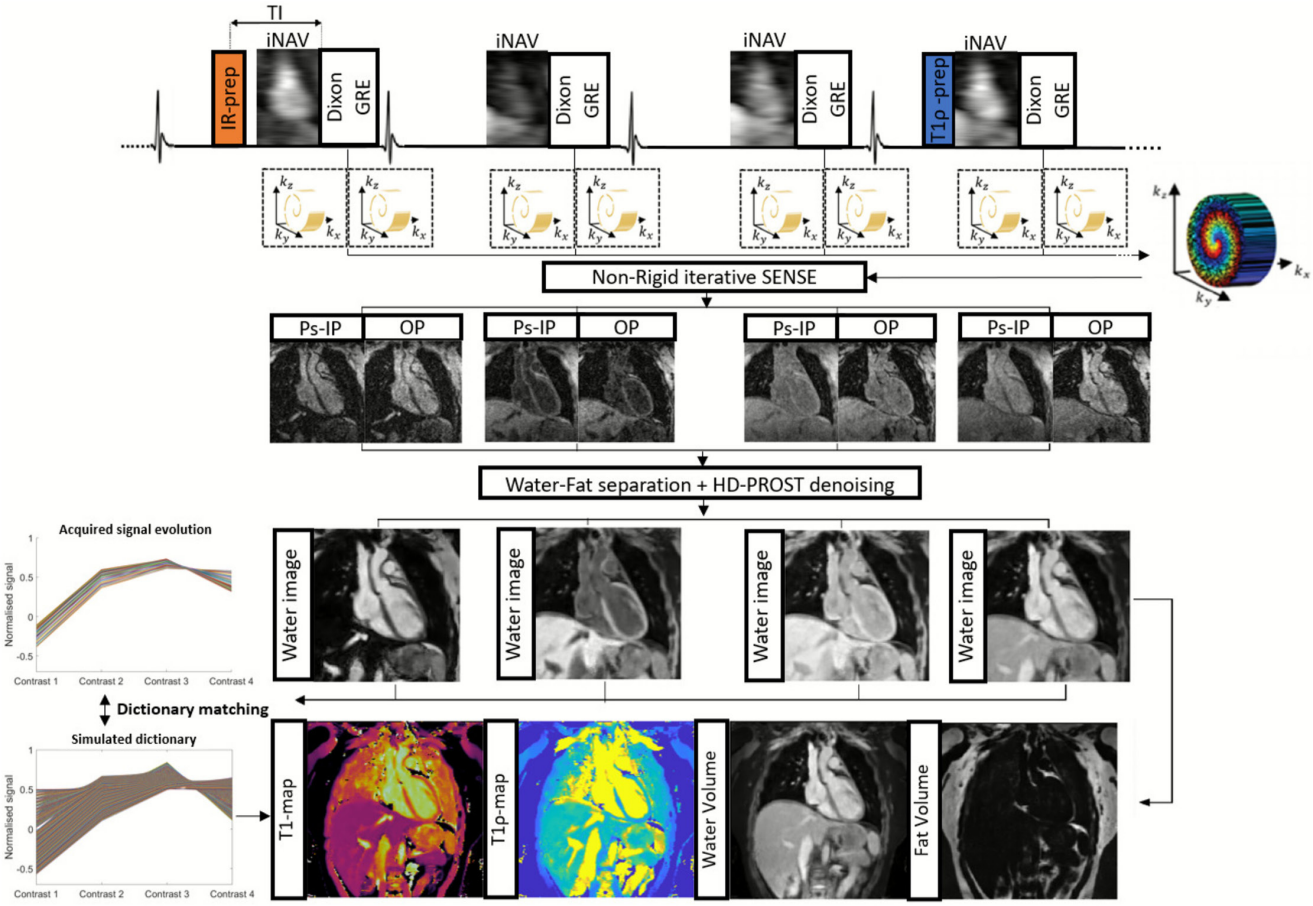
Proposed 3D joint $T_{1}$/$T_{1\rho }$ mapping and water‐fat imaging sequence framework. Four interleaved 3D volumes are acquired with preparation modules to provide $T_{1}$ and $T_{1\rho }$ encoding. Volume 1: inversion‐recovery (IR) preparation (TI=245ms), Volume 2: no‐preparation, Volume 3: no preparation, Volume 4: $T_{1\rho }$‐preparation (spin‐lock duration (TSL) =40ms, spin‐lock amplitude (SLA) =150Hz). Nonrigid motion‐corrected iterative SENSE is used to reconstruct 3D pseudo‐in‐phase (Ps‐IP)/out‐of‐phase (OP) volume images. Water and fat images are generated from Ps‐IP/OP images through a water‐fat separation algorithm, and denoised using patch‐based, multi‐dimensional low‐rank regularisation (HD‐PROST). $T_{1}$/$T_{1\rho }$ maps are estimated from water images by dictionary matching via a 1D Bloch simulation of the proposed 3D sequence.

The proposed ECG‐triggered 3D joint $T_{1}$/$T_{1\rho }$ research sequence is illustrated in Figure 1. Four acquisitions are performed in an interleaved scheme consisting of a repeating set of preparation modules defined over four heart‐beats (HBs): adiabatic inversion recovery (IR) preparation (inversion time (TI)=245ms), no preparation, no preparation and $T_{1\rho }$ preparation. No additional HBs for magnetisation recovery were used. For $T_{1\rho }$ preparation, between the tip‐down and tip‐up pulses, four spin‐locking pulses with alternating phases and two rectangular refocusing pulses with opposite phases were deployed to make $T_{1\rho }$‐preparation more robust to $B_{0}$/$B_{1}$ inhomogeneities [19]. A spin‐lock duration (TSL) =40ms and spin‐lock amplitude (SLA) =150Hz were used. Four interleaved volumes were acquired with a two‐echo bipolar Dixon RF‐spoiled gradient recalled echo (GRE) readout to acquire pseudo‐in‐phase (Ps‐IP) and out‐of‐phase (OP) echoes with TE=2.60 and 6.50ms, respectively [36]. Dixon encoding enabled fat/water separation to improve mapping quality, as well as to obtain four fat volumes that can be used for characterisation of fibrofatty infiltration of the myocardium, that has shown to be prognostically significant [38, 39]. To accelerate the acquisition, a variable‐density 3D Cartesian trajectory with spiral‐like profile order (VD‐CASPR) and golden angle step [40] was used to acquire 3D k‐space data. 2D image navigators (iNAVs) [41] were acquired prior to each 3D acquisition for beat‐to‐beat respiratory motion estimation and correction, leading to 100% respiratory scan efficiency [20]. Imaging was performed at mid‐diastole to minimise cardiac motion.

### 2.2 Image Reconstruction

#### 2.2.1 Motion‐Corrected Reconstruction

A low‐resolution 2D image in coronal orientation was generated from the 2D iNAVs at each HB. Beat‐to‐beat translational respiratory motion fields were estimated from the iNAVs, and the foot‐head translational motion field was used to assign the acquired k‐space data for each contrast into a set of B=5 respiratory bins. Intrabin 2D translational motion correction was performed by correcting the k‐space data for each bin to the same respiratory position [42]. For nonrigid motion estimation, respiratory binned images were reconstructed with iterative SENSE [42, 43] using the respiratory binned k‐space data from $1^{st}$ contrast, $1^{st}$ echo. Respiratory motion fields were estimated from the binned images through nonrigid registration with respect to the end‐expiration bin. These were used to build the following motion‐correction encoding operator, $E^{\left(c\right)}$,

(1) $$E^{\left(c\right)}=\overset{B}{\underset{b=1}{\sum }}A_{b}^{\left(c\right)}ℱSU_{b},$$

where $A_{b}^{\left(c\right)}$ is the binned sampling mask for respiratory bin b=1,…,B and contrast $c=1,\text{…},C,\;U_{b}$ the motion field for bin b,ℱ is the Fourier Transform, and S are the coil sensitivities [44]. Here, C=8 contrasts were reconstructed corresponding to the Ps‐IP and OP echoes for all four interleaved contrast acquisitions. The eight 3D image contrasts were reconstructed by solving the following optimisation problem:

(2) $$X^{\left(c\right)}=\underset{X^{\prime }}{\text{arg min}}\;||E^{\left(c\right)}X^{\prime }-K^{\left(c\right)}|{|}_{2}^{2},$$

where $X^{\left(c\right)}$ is the $c^{th}$ contrast image, $E^{\left(c\right)}$ the motion‐correction encoding operator (1) and $K^{\left(c\right)}$ the multicoil, translational motion corrected k‐space data for contrast c. This was solved using a linear conjugate‐gradient method (three iterations). Image reconstruction was performed inline with the vendor implementation following our previously developed motion‐corrected reconstruction framework [45].

#### 2.2.2 Water‐Fat Separation and Patch‐Based Low‐Rank Denoising

Water and fat images for all four interleaved contrast acquisitions were estimated from the corresponding reconstructed Ps‐IP/OP images performed inline using the vendor implementation of Dixon water‐fat separation.

The resulting four 3D water image contrasts and four 3D fat image contrasts were subsequently denoised using patch‐based, multi‐dimensional low‐rank regularisation (HD‐PROST) [37]. Parameters for the patch‐based denoising included the following: regularisation parameter λ=0.07, voxel patch‐size =5×5×5, voxel search window =20×20×20, and number of selected similar patches =20 (see [37, 45] for definitions and further details).

### 2.3 Mapping

A dictionary‐matching approach was used to estimate $T_{1}$ and $T_{1\rho }$ values from the $T_{1}$‐ and $T_{1\rho }$‐weighted water contrast images [21]. Dictionary matching was used as this has the advantage that the steady‐state signal can be numerically simulated, rather than using (approximate) analytic solutions to the Bloch equations used in exponential fitting methods. Analytic solutions are complex to derive due to the interleaved imaging and iNAV acquisitions, IR‐preparation, and $T_{1\rho }$‐preparation. A subject‐specific signal dictionary was generated through a simulation of the sequence as outlined in Section 2.3.1. The subject's heart‐rate (HR) and acquisition window were used in the simulation.

The signal polarity of reconstructed water volumes was estimated through background phase‐removal using the Ps‐IP echo images [21, 46]. For each image voxel, the polarity‐restored water images were normalised across contrasts. $T_{1}$ and $T_{1\rho }$ maps were jointly computed voxel‐by‐voxel, by selecting the parameter combination corresponding to the maximum inner‐product between the normalised voxel signal with each signal in the normalised dictionary.

#### 2.3.1 Dictionary Generation

A 1D Bloch equation simulation of the longitudinal magnetisation of the sequence was implemented to generate a signal for each volume for any $\left(T_{1},T_{1\rho }\right)$ parameter combination. It is assumed that the proposed sequence, which deploys both RF‐spoiling and gradient‐spoiling for the 3D imaging and iNAV readouts and gradient spoiling immediately after the IR‐prep and $T_{1\rho }$‐prep pulses, completely eliminates transverse magnetisation justifying the use of a simulation of the longitudinal magnetisation only. Simulations were performed using the subject's HR and acquisition window. Each signal evolution in the dictionary (corresponding to a unique parameter combination) was normalised. The dictionary used the following parameter combinations ([start:step:end]): $\begin{array}{c} T_{1}=[50:5:1400,1400:50:1800,1800:100:3000]\;\text{ms},\; \end{array}\begin{array}{c} T_{1\rho }=[5:5: \end{array}\begin{array}{c} 20,20:1:80,80:4:300,300:100:600]\;\text{ms} \end{array}$.

### 2.4 Experiments

The proposed 3D joint $T_{1}$/$T_{1\rho }$ sequence was tested in numerical simulations, phantoms, healthy subjects, and a patient. Data were acquired on a 0.55‐T MR scanner (MAGNETOM Free.MAX, Siemens Healthineers AG, Erlangen, Germany) with a six‐channel chest array and nine‐channel spine array, with an external ECG monitor (Expression MR400, Philips Healthcare, Best, Netherlands). Written informed consent was obtained from all participants before undergoing the MR scan, and the study was approved by the institutional review board.

#### 2.4.1 Simulations

Numerical simulations of the proposed sequence were performed to test the accuracy and precision of the sequence. For any parameter combination $\left(T_{1},T_{1\rho }\right)$, corresponding to a different tissue, a Bloch simulation of the sequence was performed as described in Section 2.3.1 to create a signal for each volume. White Gaussian noise with constant variance/volume was pseudo‐randomly generated and added to the signal for each volume, and the noisy signal evolution matched to the dictionary. This was repeated over $N_{n}$ noise trials to generate $N_{n}$ estimated parameters ${\left\{(T_{1},T_{1\rho })\right\}}_{i}$ $i=1,\text{…},N_{n}$. The bias/coefficient of variation (CV) were then estimated as

(3) $$\text{Bias}(Y)=\mu_{Y}-Y,\text{CV}(Y)=\sigma_{Y}/\mu_{Y},$$

where Y= $T_{1}$ or $T_{1\rho }$ and $\mu_{Y},\;\sigma_{Y}$ are the mean and standard deviation (SD) of Y respectively. This process was repeated for each tissue of interest ${\left\{(T_{1},T_{1\rho })\right\}}_{p},\;p=1,\text{…},N_{p}$.

For the simulations, the parameter space of interest was selected as $T_{1}=[500:50:1400]\;\text{ms},\;T_{1\rho }=[40:4:80]\;\text{ms}$ matching typical myocardial tissue values at 0.55 T. For each parameter combination, $N_{n}=10000$ noise trials were used, with standard deviation of noise $=\frac{1}{350}M_{0}$. Simulated HRs in the range [60:20:120]bpm with an acquisition window =155 ms were used.

#### 2.4.2 Phantoms

Data were acquired in the standardised $T_{1}$‐MES phantom [47] (9 vials, ≈30mm diameter) and an in‐house $T_{1}/T_{1\rho }$ phantom (10 vials, ≈28mm diameter) with variable ${\text{NiCl}}_{2}$ ($T_{1}$) and agar ($T_{1\rho }$) concentration [48]. In the in‐house $T_{1}/T_{1\rho }$ phantom, the ${\text{NiCl}}_{2}$ concentration varied from 0 to 5.02 mM and the agar concentration varied from 0.5–4.5 w/w. Data were acquired with the proposed 3D joint $T_{1}$/$T_{1\rho }$ mapping sequence, 2D IR spin‐echo (SE), 2D $T_{1\rho }$‐prep SE, research 2D modified Look‐Locker inversion recovery (MOLLI) [11] and research 2D $T_{1\rho }$ [19] reference sequences.

Data were also acquired in an in‐house water‐fat phantom (eight vials, ≈28mm diameter, with variable concentration (0%–100%) of peanut oil [49]). Data were acquired with the proposed 3D joint $T_{1}$/$T_{1\rho }$ mapping sequence and a 3D two‐echo VIBE [50] Dixon reference sequence.

The proposed 3D joint $T_{1}$/$T_{1\rho }$ mapping was acquired in transverse orientation, TI=245ms, two‐echo bipolar Dixon GRE (${\text{TE}}_{1}/{\text{TE}}_{2}/\text{TR}=2.60/6.50/9.71\;\text{ms}$, flip angle (FA) $=8^{\circ }$, bandwidth (BW) =451Hz/pixel), iNAV (14(×2)‐echoes, FA $=3^{\circ }$), VD‐CASPR acceleration ×4 with centric k‐space reordering, field of view (FOV) $=320\times 320\times 20\;{\text{mm}}^{3}$ and resolution =2mm isotropic. An acquisition window =155ms was used corresponding to 16 segments acquired per HB.

For 2D IR SE, imaging parameters included TE/TR=12/8000ms, FA $=90^{\circ }$, resolution $=2.3\times 2.3\;{\text{mm}}^{2}$, slice‐thickness =10mm, BW =130Hz/pixel. 8 IR‐preps with TIs=[50,350,650,950,1250,1550,1850,2150]ms were used. A two‐parameter fit, $\left(T_{1},M_{0}\right)$, to the following signal equation $S(T_{1},M_{0},\text{TI},\text{TR})=M_{0}(1-2\exp(-\text{TI}⁄T_{1})+\exp(-\text{TR}⁄T_{1}))$ was used, solved using Levenberg–Marquardt algorithm.

For 2D $T_{1\rho }$‐prep SE, imaging parameters included TE/TR=12/5000ms, FA $=90^{\circ }$, resolution $=2.3\times 2.3\;{\text{mm}}^{2}$, slice‐thickness =10mm, BW =130Hz/pixel. 8 $T_{1\rho }$‐preps with TSL =[2,14,26,38,50,62,74,86]ms and SLA=150Hz were used. $T_{1\rho }$ was obtained through linear least‐squares fitting to the log of the following signal equation, $S(T_{1\rho },M_{0},\text{TSL})=M_{0}\exp(-\text{TSL}/T_{1\rho })$.

The 2D MOLLI 5(3)3 sequence was deployed with bSSFP readout, FA $=50^{\circ }$, resolution $=2.3\times 2.3\;{\text{mm}}^{2}$, slice‐thickness =10mm,TE/TR=1.89/4.45ms, BW =539Hz/pixel. The $T_{1}$ weighted images and maps were obtained inline on the scanner.

The 2D $T_{1\rho }$ reference sequence was ECG‐triggered and consisted of acquisition of 4 $T_{1\rho }$‐prepared images with TSL =[2,15,30,40]ms and SLA=150Hz. The acquisition of each $T_{1\rho }$‐weighted contrast image was segmented over 3 HBs, resulting in a total acquisition time of 12 HBs per short‐axis $T_{1\rho }$ map. A saturation (SAT) pulse was applied at every HB, with the trigger delay chosen to give a constant duration between the SAT pulse and beginning of $T_{1\rho }$‐preparation, to ensure $M_{z}$ was the same prior to each $T_{1\rho }$‐preparation avoiding additional HBs for magnetisation recovery [19]. A two‐echo Dixon GRE (${\text{TE}}_{1}/{\text{TE}}_{2}/\text{TR}=2.60/6.24/7.38\;\text{ms}$, FA $=20^{\circ }$, BW =453Hz/pixel) was used, with parallel imaging using GRAPPA (acceleration factor ×2), centric k‐space reordering, FOV $=300\times 300\;{\text{mm}}^{2}$, resolution $=2\times 2\;{\text{mm}}^{2}$, slice‐thickness =15mm and a fixed 199ms acquisition window. $T_{1\rho }$‐weighted contrast images were obtained inline on the scanner, followed by HD‐PROST denoising. Mono‐exponential fitting was used to generate a $T_{1\rho }$ map from the $T_{1\rho }$‐weighted water images.

For 3D two‐echo VIBE Dixon reference sequence, imaging parameters included the following: 3D non–ECG‐gated CAIPIRINHA, stack of stars k‐space sampling, FOV = $379\times 325\times 240\;{\text{mm}}^{3}$, resolution $=0.85\times 0.85\times 3\;{\text{mm}}^{3}$, FA $=20^{\circ }$, and ${\text{TE}}_{1}/{\text{TE}}_{2}/\text{TR}=3.06/6.47/9.74\;\text{ms}$.

##### 2.4.2.1 Data Analysis

Circular regions of interest (ROI) covering the centre of each vial of ≈20mm diameter were manually drawn on each phantom. For the $T_{1}$‐MES and in‐house $T_{1}/T_{1\rho }$ phantom, the mean and CV of $T_{1}$/$T_{1\rho }$ mapping values obtained with the proposed approach, $T_{1}$ obtained with 2D IR SE and $T_{1\rho }$ obtained with 2D $T_{1\rho }$‐prep SE were measured for each vial. Sum of squared differences (SSD) were calculated between the measured signal and simulated signal for the proposed approach, and mean SSD was measured for each vial. Agreement between $T_{1}/T_{1\rho }$ mapping values obtained with the proposed approach and SE were assessed through linear regression and Bland–Altman analysis. Vials with $T_{1}\leq 1300\;\text{ms}$ and $T_{1\rho }\leq 150\;\text{ms}$ were selected prior to fitting to focus on the typical myocardial tissue value range.

For the in‐house water‐fat phantom, the mean and CV of fat signal values obtained from the third contrast with the proposed approach and 3D two‐echo VIBE Dixon research reference sequence were measured for each vial. Agreement between fat signal values obtained with the proposed approach and reference was assessed through linear regression and Bland–Altman analysis.

#### 2.4.3 In Vivo Study

Data were acquired in 11 healthy subjects (seven male, age 31±4 years). For each subject, data were acquired with the proposed 3D joint $T_{1}$/$T_{1\rho }$ mapping sequence. For comparison purposes, data were acquired with research 2D MOLLI [11] and research 2D $T_{1\rho }$ [19] mapping sequences at mid‐ventricular short‐axis (SAx) slice for each healthy subject, as well as research 3D VIBE Dixon reference in four healthy subjects.

For the proposed 3D joint $T_{1}$/$T_{1\rho }$ mapping, after acquisition of a cardiac localiser, a subject‐specific slice selection covering the heart was planned with FOV $=320\times 320\times 96-120\;{\text{mm}}^{3}$. The sequence was performed in coronal orientation. A subject‐specific trigger‐delay and acquisition window were informed after acquisition of a free‐breathing four‐chamber CINE. The acquisition window was 155ms. The remaining imaging parameters matched those used for phantoms. The total acquisition time was 11.0±2.9 min. For the proposed 3D joint $T_{1}$/$T_{1\rho }$ reconstruction, the computational time was ≈5min for nonrigid iterative SENSE reconstruction and water/fat separation (implemented inline), ≈6min for HD‐PROST denoising of water and fat images (implemented in MATLAB (The MathWorks Inc, Natick, MA, USA) calling a C++ library) and ≈3min for dictionary generation and matching (implemented in MATLAB), with a total reconstruction time of ≈15min. Reconstructions were performed using an Intel(R) Core(TM) i7‐9750H CPU (2.60 GHz) and 32 GB RAM.

The 2D MOLLI and 2D $T_{1\rho }$ reference sequences were acquired during a breath‐hold at mid‐diastole. The remaining imaging parameters matched those used in phantom experiments.

The 3D VIBE Dixon reference was acquired under breath‐hold at end‐expiration. The remaining imaging parameters matched those used in phantom experiments.

The proposed 3D joint $T_{1}$/$T_{1\rho }$ was also acquired in one patient with acute myocarditis. Acquisition parameters matched those used for healthy subjects. Additionally, a 2D PSIR LGE research sequence (resolution $=1.7\times 1.7\;{\text{mm}}^{2}$, slice‐thickness =8mm, FA $=80^{\circ }$) was acquired under breath‐hold in several SAx views.

##### 2.4.3.1 Data Analysis

The proposed 3D joint $T_{1}$/$T_{1\rho }$ maps were reformatted in the SAx view to match the 2D MOLLI and 2D $T_{1\rho }$ midventricular SAx slice. For all subjects, a 16‐segment AHA model [51] was calculated on the reformatted SAx slices of both the reconstructed 3D $T_{1}$ and $T_{1\rho }$ maps. For all subjects, SSD maps were also calculated and a 16‐segment AHA model generated. Mean, SD and CV were calculated for each segment of the AHA model. 2D MOLLI and 2D $T_{1\rho }$ were also segmented, and mean, SD and CV were calculated for the midventricular segments of the AHA model.

The accuracy and precision of mapping values obtained with the proposed 3D joint $T_{1}$/$T_{1\rho }$ mapping were compared to research 2D MOLLI [11] and research 2D $T_{1\rho }$ [19] mapping sequences at midventricular SAx slice. Paired sample t‐tests (p=0.05) were used to analyse differences in mean/CV $T_{1}$/$T_{1\rho }$ values.

## 3 Results

### 3.1 Simulations

**FIGURE 2:**
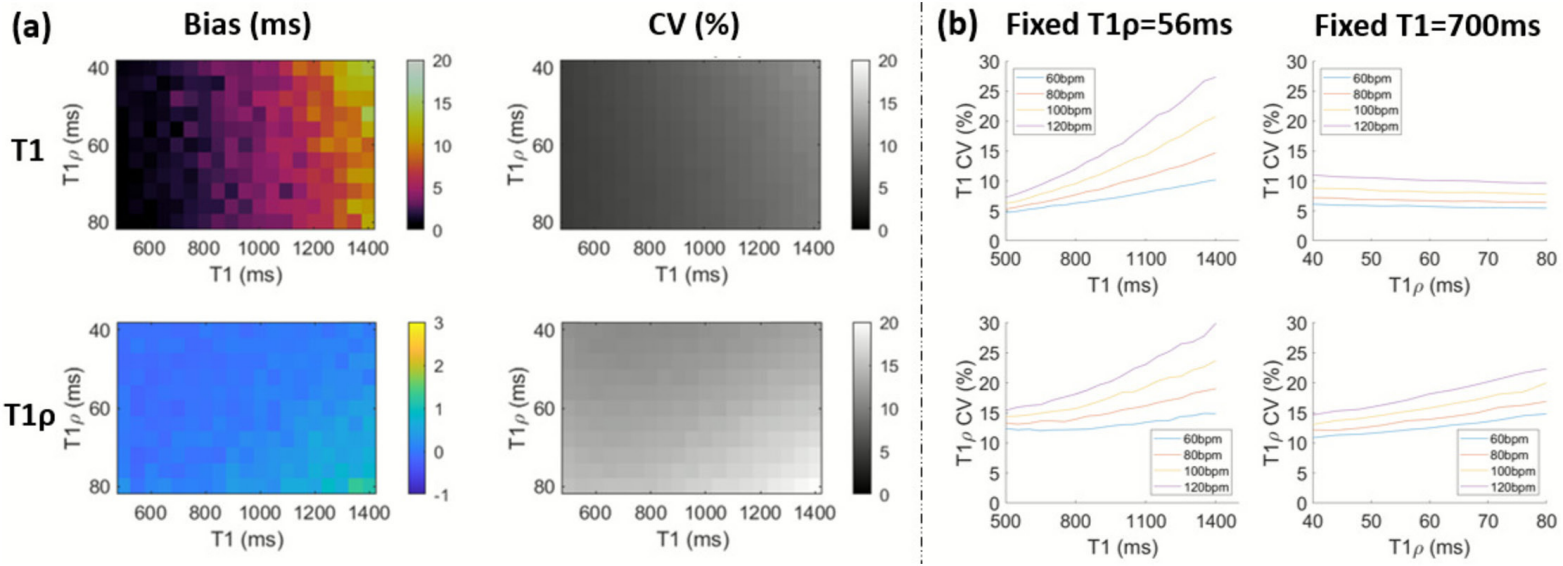
(a) Simulation of sequence accuracy and precision over a range of $T_{1}$/$T_{1\rho }$ values with simulated HR =60bpm. Left/Right are Bias (ms) and coefficient of variation (CV) (%), and Top/Bottom are $T_{1}$/$T_{1\rho }$ values. (b) Simulation of sequence precision over a range of simulated HR=[60:20:120]bpm. Top and Bottom are $T_{1}$ and $T_{1\rho }$ CV, and Left and Right are CV plotted against $T_{1}$ (fixed $T_{1\rho }=56\;\text{ms}$) and $T_{1\rho }$ (fixed $T_{1}=700\;\text{ms}$) respectively.

Figure 2 indicates a plot of Bias and CV for HR =60bpm, which demonstrated generally low bias and CV (<21%) for both $T_{1}$ and $T_{1\rho }$ over the parameter range simulated. $T_{1}$ and $T_{1\rho }$ CV are also plotted as a function of $T_{1}$ (fixed $T_{1\rho }=56\;\text{ms}$) and $T_{1\rho }$ (fixed $T_{1}=1100\;\text{ms}$) over a range of HRs=[60:20:120]bpm. First, it was observed that the sequence is more precise for both $T_{1}$ and $T_{1\rho }$ for shorter $T_{1}$. Also, increased HR increases $T_{1}$ and $T_{1\rho }$ CV, but the increase in CV over HR is smaller for shorter $T_{1}$ and thus should be a lesser issue at low‐field due to the reduction of $T_{1}$ vs 1.5T. For typical myocardial tissue ($T_{1}=700\;\text{ms},\;T_{1\rho }=56\;\text{ms}$), CV was <18% over all simulated HRs.

### 3.2 Phantoms

**FIGURE 3:**
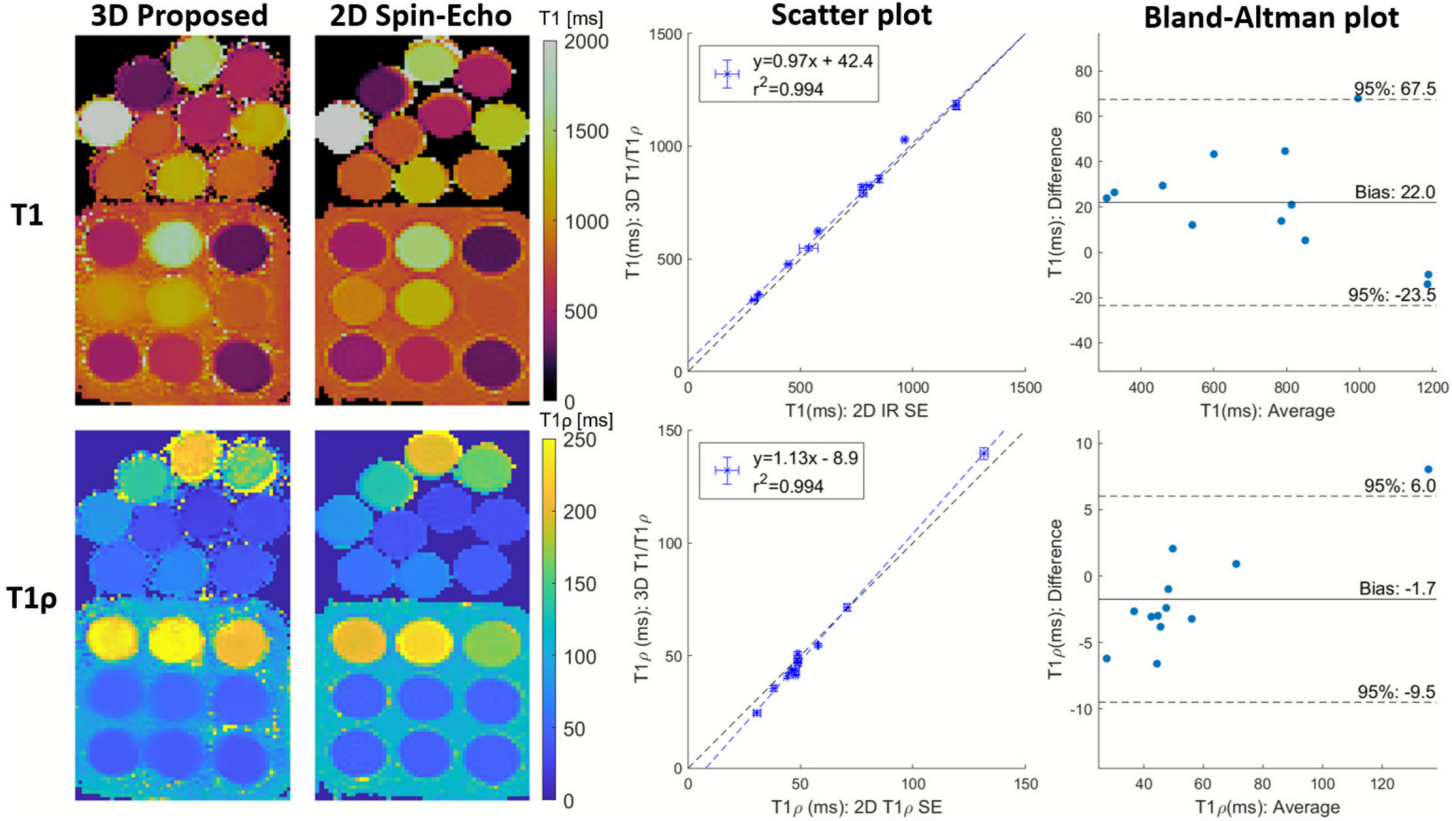
Left: Comparison of proposed 3D joint $T_{1}$/$T_{1\rho }$ mapping to 2D inversion‐recovery (IR) spin‐echo (SE) and 2D $T_{1\rho }$‐prep SE (simulated heart‐rate (HR)=60 bpm). The top 10 vials are from the in‐house $T_{1}$/$T_{1\rho }$ phantom, and the bottom 9 vials are from the $T_{1}$‐MES phantom. Middle: Scatter plot comparison of proposed 3D joint $T_{1}$/$T_{1\rho }$ to 2D IR SE and 2D $T_{1\rho }$‐prep SE. The black and blue lines are identity lines and linear fits, respectively. Right: Bland–Altman plot comparison of proposed 3D joint $T_{1}$/$T_{1\rho }$ to 2D IR SE and 2D $T_{1\rho }$‐prep SE.

A comparison of the proposed 3D joint $T_{1}$/$T_{1\rho }$ maps to 2D SE references in phantoms with a simulated HR=60 bpm is shown in Figure 3. When comparing the 3D joint $T_{1}$/$T_{1\rho }$ sequence to 2D IR SE and 2D $T_{1\rho }$‐prep SE, high linear correlations of y=0.97x+42.4 ($r^{2}=0.994)$ and y=1.13x−8.9 ($r^{2}=0.994)$ were observed, respectively, with a bias of 22 and −1.7ms for $T_{1}$ and $T_{1\rho }$, respectively. The proposed 3D joint $T_{1}$/$T_{1\rho }$ mapping sequence was compared with 2D SE references over a range of simulated HRs (Figure S1). The proposed 3D joint $T_{1}$/$T_{1\rho }$ mapping sequence fat signal of the third contrast was compared to the 3D VIBE Dixon research sequence in the in‐house water‐fat phantom (Figure S2). A phantom experiment with heart‐rate variability was also performed (Figure S3). The proposed 3D joint $T_{1}$/$T_{1\rho }$ mapping was compared to 2D MOLLI and 2D $T_{1\rho }$ reference sequences in the T1‐MES and in‐house $T_{1}/T_{1\rho }$ phantoms (Figure S4). The proposed 3D joint $T_{1}$/$T_{1\rho }$ and SSD maps for the T1‐MES and in‐house $T_{1}/T_{1\rho }$ phantoms are also shown in Figure S5.

### 3.3 In Vivo Study

**FIGURE 4:**
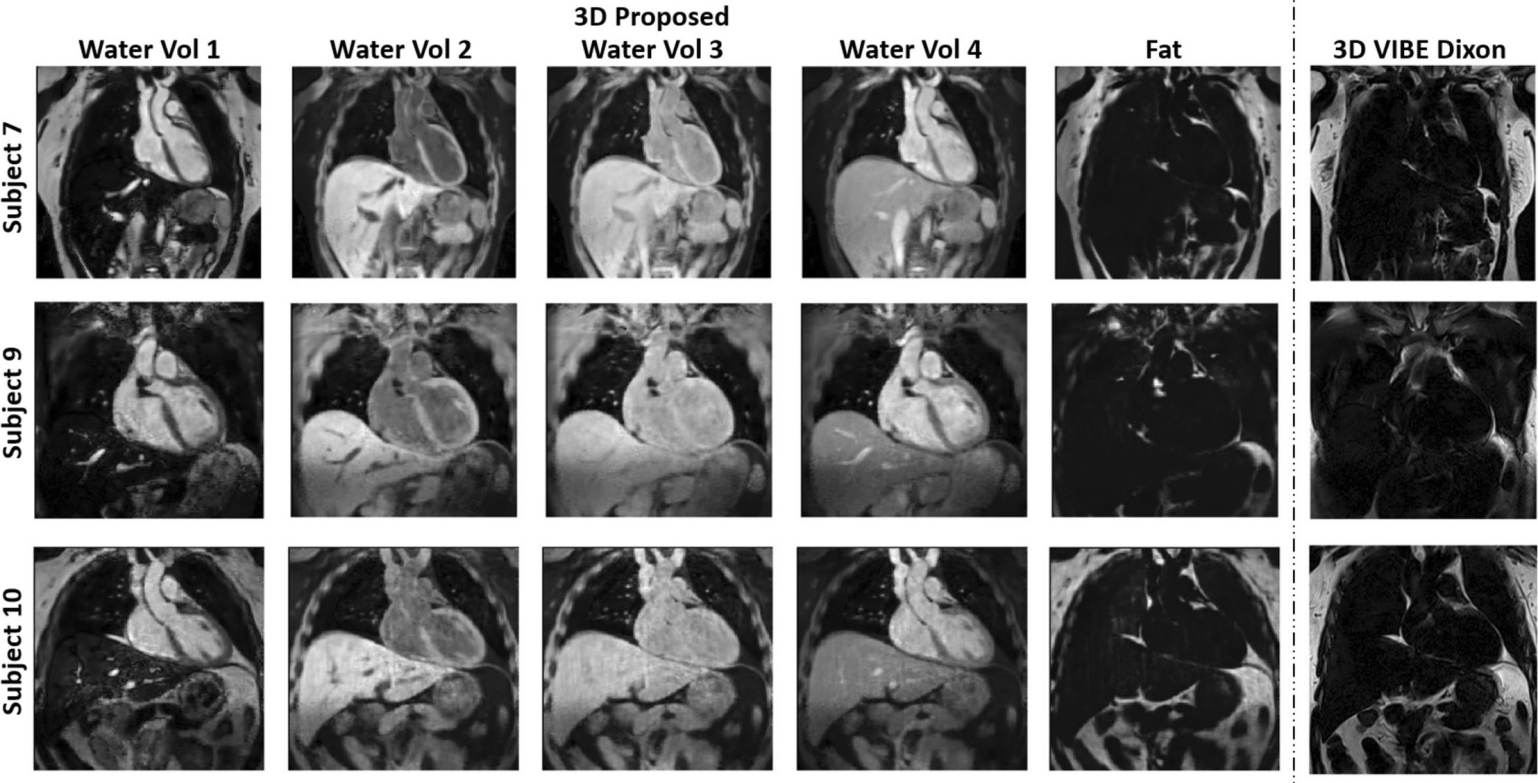
3D water images for each volume (vol) and fat image (third volume) from the proposed 3D joint $T_{1}$/$T_{1\rho }$ mapping sequence with Dixon encoding in coronal view for 3 representative healthy subjects. The fat images from the 3D VIBE Dixon reference are included in the right column for comparison.

**FIGURE 5:**
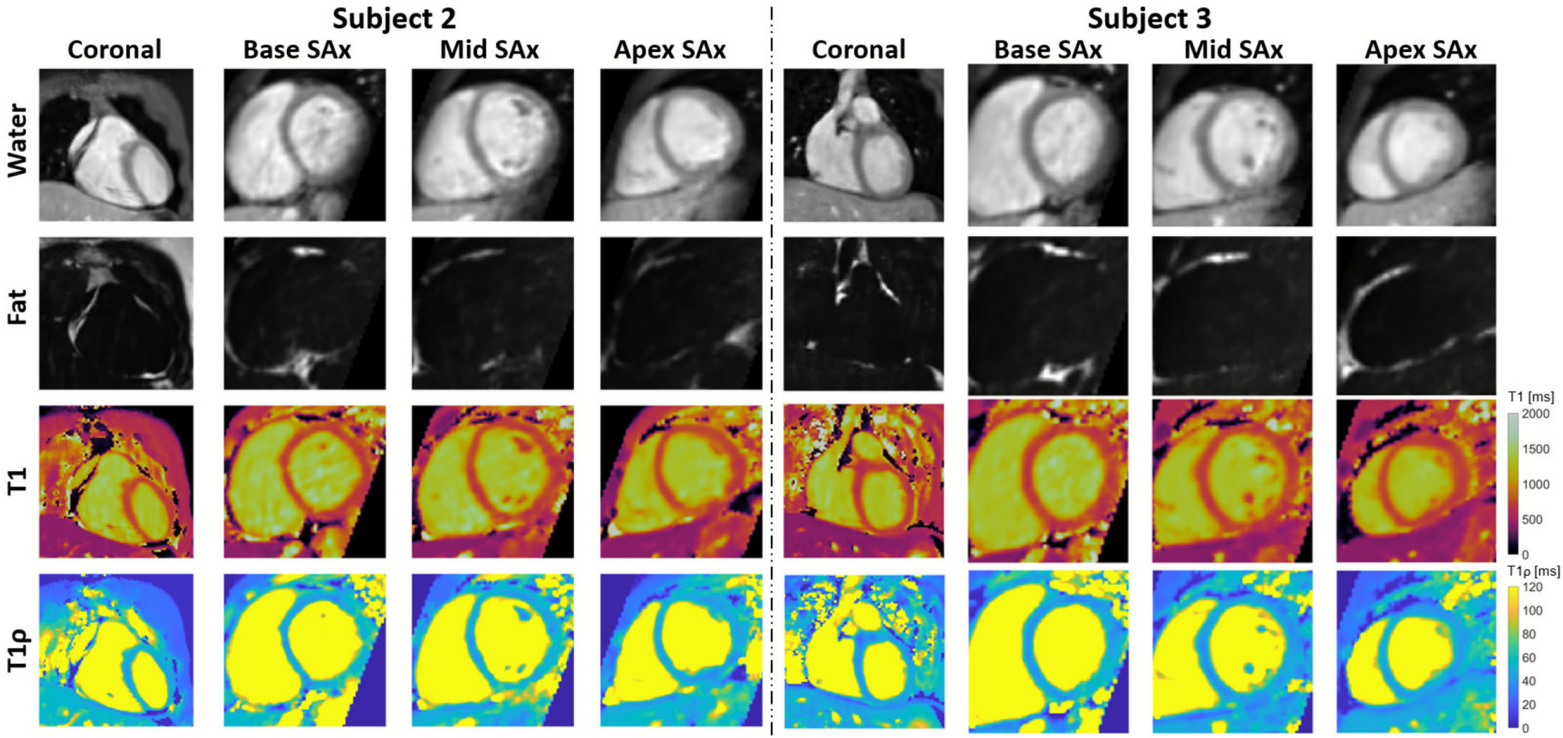
Water (fourth volume), fat (third volume), $T_{1}$ and $T_{1\rho }$ mappings from the proposed 3D joint $T_{1}$/$T_{1\rho }$ mapping sequence with Dixon encoding showing coronal, base/mid/apex‐ventricular short‐axis (SAx) views through the 3D reconstruction in 2 representative healthy subjects.

Water contrast images across all four contrast interleaved acquisitions of the proposed sequence for three representative subjects are shown in Figure 4. The fat image (third contrast) of the proposed sequence and fat image of the 3D VIBE Dixon reference are also included. Good quality water contrast images were observed, and good qualitative comparison was observed between the proposed sequence fat image and the 3D VIBE Dixon reference. Different views of the 3D volume of water (fourth contrast), fat (third contrast) and $T_{1}$ and $T_{1\rho }$ maps for two representative healthy subjects are shown in Figure 5. Uniform myocardial $T_{1}$ and $T_{1\rho }$ quantification can be seen in different views.

**FIGURE 6:**
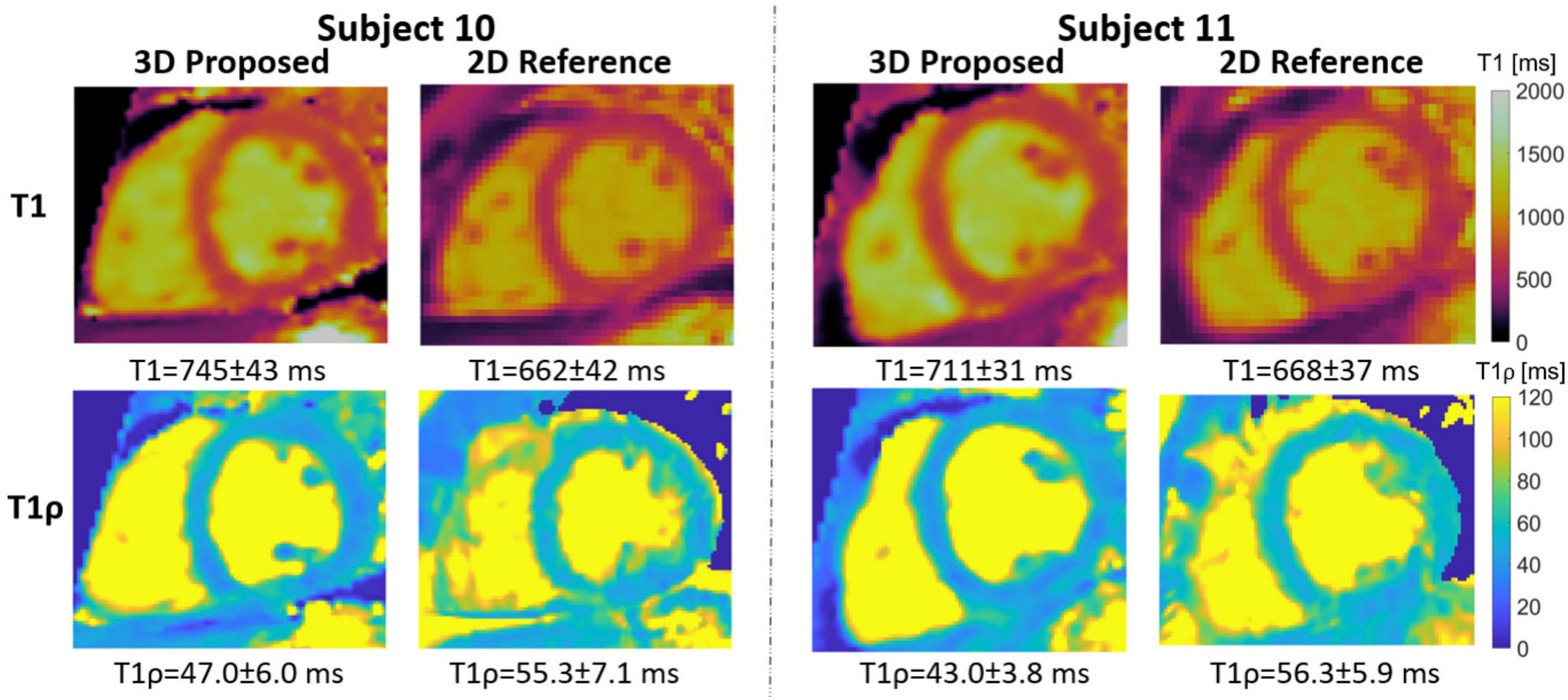
Comparison of proposed 3D joint $T_{1}$/$T_{1\rho }$ mapping to 2D MOLLI and 2D $T_{1\rho }$ for 2 representative healthy subjects. Mean and standard deviation of mid‐ventricular septal myocardial values are displayed.

**FIGURE 7:**
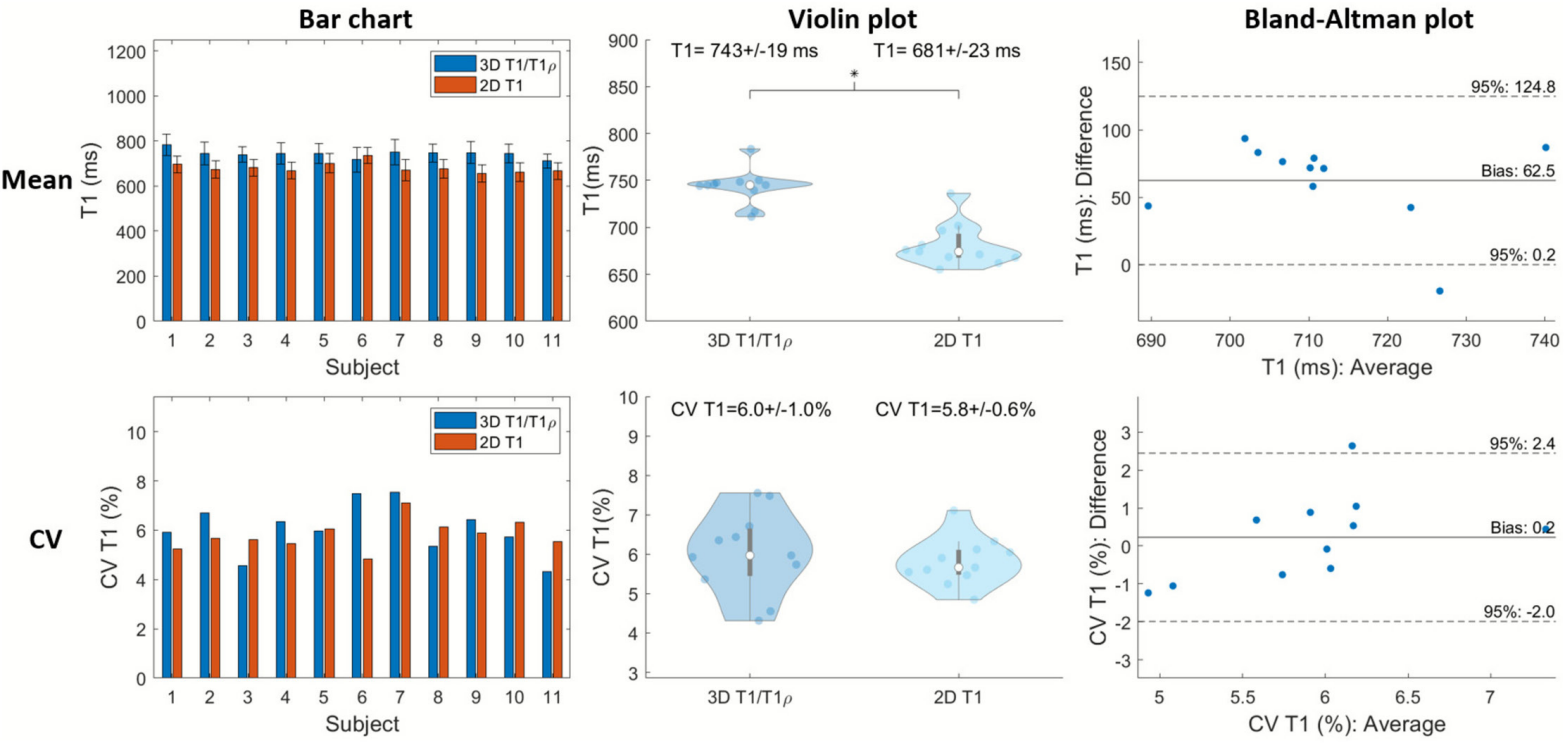
Comparison of mid‐ventricular short‐axis septal myocardium values for proposed $T_{1}$ and 2D MOLLI across healthy subjects. Top/Bottom figure are mean/coefficient of variation (CV) of myocardial values. Left/Middle/Right are bar chart/violin plot/Bland‐Altman plot. Asterisks placed over the violin plots indicate significant differences (p<0.05).

**FIGURE 8:**
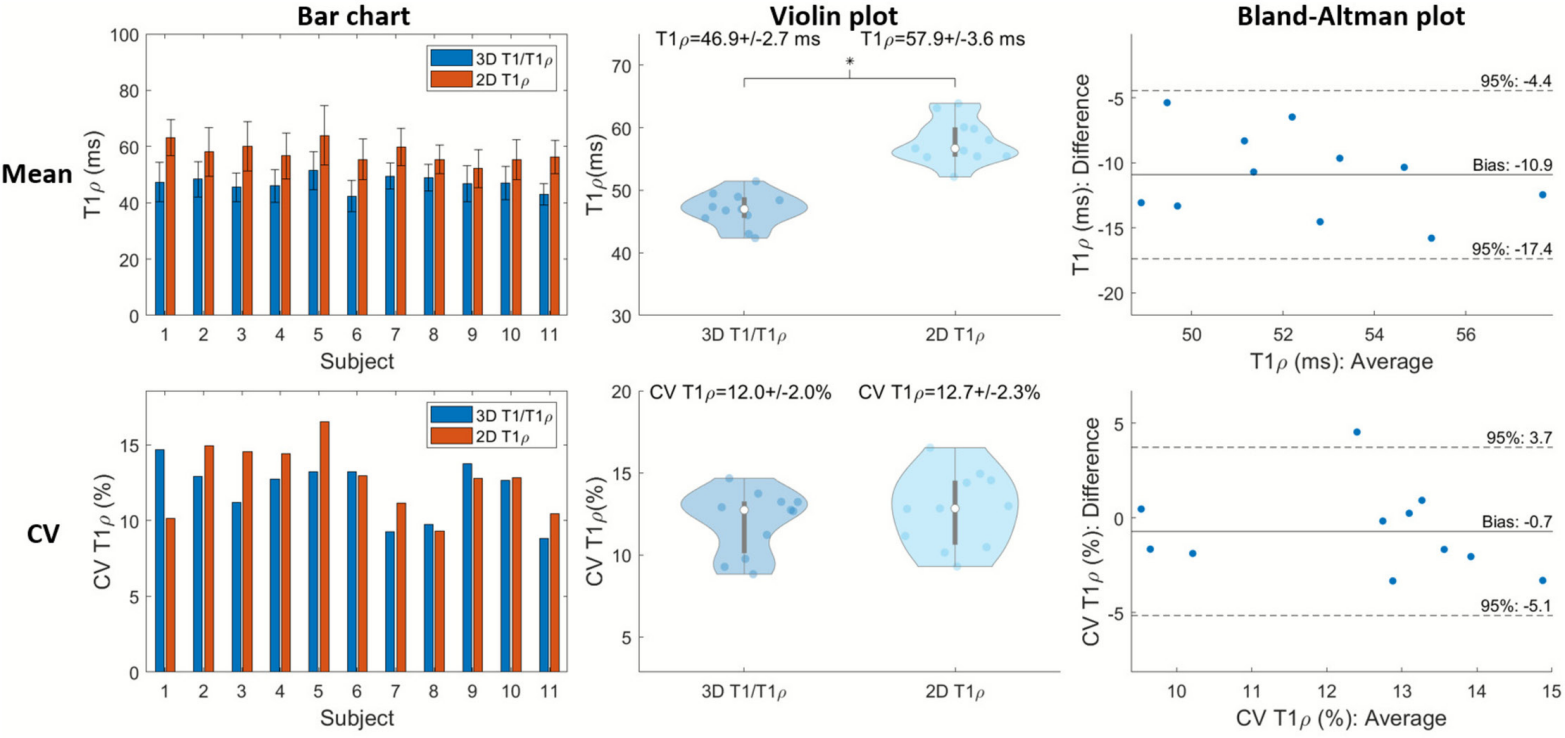
Comparison of mid‐ventricular short‐axis septal myocardium values for proposed $T_{1\rho }$ and 2D $T_{1\rho }$ across healthy subjects. Top/Bottom figure are mean/coefficient of variation (CV) of myocardial values. Left/Middle/Right are bar chart/violin plot/Bland‐Altman plot. Asterisks placed over the violin plots indicate significant differences (p<0.05).

Figure 6 illustrates midventricular SAx views of the proposed 3D joint $T_{1}$/$T_{1\rho }$ maps, 2D MOLLI and 2D $T_{1\rho }$ for two representative healthy subjects. The proposed 3D joint $T_{1}$/$T_{1\rho }$ and SSD maps are also shown for a representative healthy subject (Figure S5). A comparison of midventricular SAx septal myocardium values measured with the proposed and reference sequences across all healthy subjects is illustrated in Figures 7 and 8 for $T_{1}$ and $T_{1\rho }$, respectively. Comparing midventricular SAx septal myocardium values, mean $T_{1}$ for the proposed 3D joint $T_{1}$/$T_{1\rho }$ sequence (743±19ms) was significantly larger (p<0.0001) than 2D MOLLI (681±23ms). CV $T_{1}$ for the proposed sequence (6.0%±1.0%) was slightly larger than 2D MOLLI (5.8%±0.6%) but not significantly (p=0.52). Mean $T_{1\rho }$ for the proposed 3D joint $T_{1}$/$T_{1\rho }$ sequence (46.9±2.7ms) was significantly smaller (p<0.0001) than 2D $T_{1\rho }$ (57.9±3.6ms). CV $T_{1\rho }$ for the proposed sequence (12.0%±2.0%) was slightly smaller than 2D $T_{1\rho }$ (12.7%±2.3%) but not significant (p=0.32).

**FIGURE 9:**
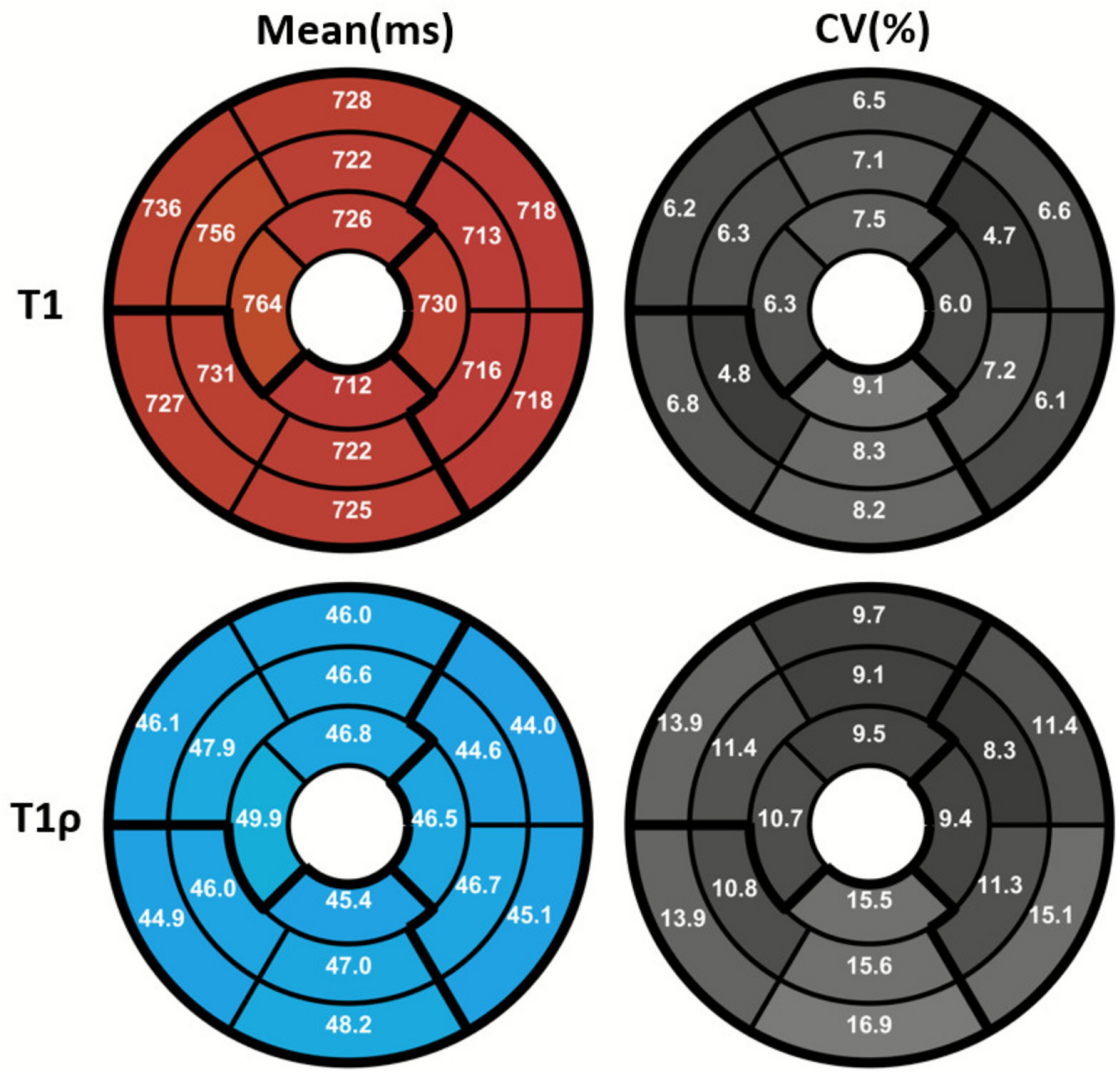
Bull's eye plots of mean and coefficient of variation (CV) for $T_{1}$ and $T_{1\rho }$ for proposed 3D joint $T_{1}$/$T_{1\rho }$ mapping averaged across all 11 healthy subjects.

Figure 9 shows bull's eye plots of mean and CV for $T_{1}$ and $T_{1\rho }$ for the proposed 3D joint $T_{1}$/$T_{1\rho }$ mapping sequence averaged across all healthy subjects, demonstrating uniform mean $T_{1}$ and $T_{1\rho }$ values over the 16 AHA segments. Violin plots of mean and CV for $T_{1}$ and $T_{1\rho }$ for each AHA segment across all subjects are also shown in Figure S6. Mean/CV $T_{1}$ and $T_{1\rho }$ values for different regions of the heart averaged across all healthy subjects are tabulated in Table S1. Left‐ventricle myocardium values for the proposed sequence averaged across all healthy subjects were computed to be $T_{1}$ mean/CV =728±20 ms/6.7%±0.7% and $T_{1\rho }$ mean/CV =46.3±2.3 ms/12.1%±1.2%.

Mean $T_{1}$ in base, mid and apex segments were calculated to be 725±19, 727±23, and 733±24ms, respectively. CV $T_{1}$ in base, mid and apex segments were calculated to be 6.7%±1.2%,6.4%±0.9% and 7.2%±1.5%, respectively. There was no statistically significant differences for mean or CV $T_{1}$ between these segments. Mean $T_{1\rho }$ in base, mid and apex segments were calculated to be 45.7±2.8, 46.5±2.3 and 47.1±2.6 ms, respectively. CV $T_{1\rho }$ in base, mid and apex segments were calculated to be 13.6%±2.5%,11.1%±1.4% and 11.2%±1.8%, respectively. CV base $T_{1\rho }$ was significantly higher than CV mid $T_{1\rho }$ (p=0.03) and CV apex $T_{1\rho }$ (p=0.01), and there was no statistically significant differences for mean or CV $T_{1\rho }$ between other segments.

Mean $T_{1}$ in anterior, inferior, septal and lateral segments were calculated to be 725±24, 720±33, 743±19 and 719±29 ms, respectively. CV $T_{1}$ in anterior, inferior, septal and lateral segments were calculated to be 7.0%±1.4%,8.6%±1.9%,6.1%±0.8% and 6.1%±1.0%, respectively. Comparing mean and CV $T_{1}$ between anterior and inferior or septal and lateral segments, mean lateral $T_{1}$ was significantly shorter than mean septal $T_{1}$ (p=0.02), and there were no statistically significant differences between other segments. Mean $T_{1\rho }$ in anterior, inferior, septal and lateral segments were calculated to be 46.4±3.0, 46.9±4.8, 46.9±2.7 and 45.4±2.7 ms, respectively. CV $T_{1\rho }$ in anterior, inferior, septal and lateral segments were calculated to be 9.4%±1.3%,16.2%±3.2%,12.1%±1.6% and 11.1%±1.8%, respectively. Comparing mean and CV $T_{1\rho }$ between anterior and inferior or septal and lateral segments, CV inferior $T_{1\rho }$ was significantly higher than CV anterior $T_{1\rho }$ (p<0.001) and there were no statistically significant differences between other segments.

**FIGURE 10:**
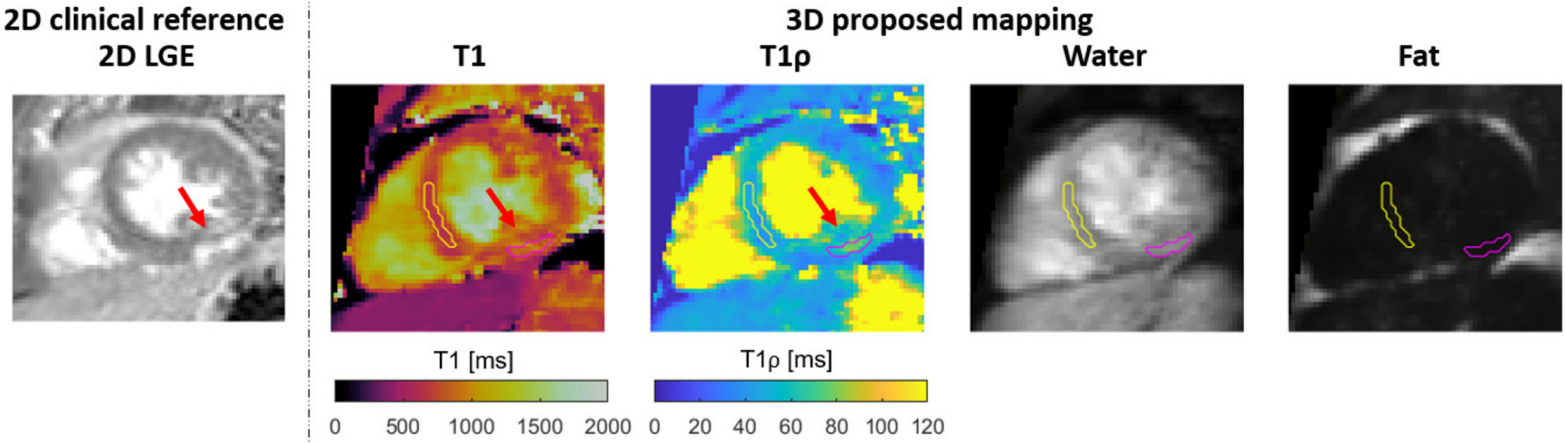
Comparison of proposed 3D joint $T_{1}$/$T_{1\rho }$ mapping with 2D LGE for a patient with acute myocarditis. 3D water (fourth volume) and fat (fourth volume) images are included for reference. Increased myocardial signal intensity can be observed in the midventricular inferolateral wall of the clinical 2D LGE image. The proposed 3D joint $T_{1}$/$T_{1\rho }$ mapping sequence indicates elevated $T_{1}$ and $T_{1\rho }$ in the scar region (pink region of interest [ROI]) relative to remote (yellow ROI). Remote and scar $T_{1}$ values were estimated to be 675±34 and 872±87ms, respectively. Remote and scar $T_{1\rho }$ values were estimated to be 44.7±4.6 and 66.1±9.8ms, respectively.

Finally, we present results from a patient with suspected acute myocarditis in Figure 10. Subepicardial enhancement extending transmurally in the midventricular inferolateral wall was observed in the 2D LGE. The proposed 3D joint $T_{1}$/$T_{1\rho }$ mapping indicated elevated $T_{1}$ and $T_{1\rho }$ values in the corresponding area in the mid inferolateral wall (yellow and pink ROIs indicate the remote and scar regions, respectively). Remote and scar $T_{1}$ values were estimated to be 675±34 and 872±87ms, respectively. Remote and scar $T_{1\rho }$ values were estimated to be 44.7±4.6 and 66.1±9.8ms, respectively. 3D water (fourth volume) and fat (fourth volume) images of the proposed sequence were included for reference.

## 4 Discussion

A novel free‐breathing, 3D whole‐heart joint $T_{1}$/$T_{1\rho }$ mapping sequence with Dixon encoding has been proposed to provide coregistered 3D $T_{1}$ and $T_{1\rho }$ maps and water‐fat volumes with isotropic spatial resolution in a single ≈11‐min scan for comprehensive contrast‐agent free myocardial tissue characterization and visualisation of the whole‐heart anatomy at 0.55 T. The proposed sequence is inspired by a recent 3D joint $T_{1}$/$T_{2}$ sequence proposed at 1.5T [21], but with $T_{2}$‐preparation replaced by $T_{1\rho }$‐preparation [52], the two‐point Dixon echo times modified to acquire a Ps‐IP echo (instead of the first IP echo) to minimise scan‐time following Nayak et al. [36] and migrated to 0.55 T. $T_{2}$‐preparation was replaced with $T_{1\rho }$‐preparation since $T_{2}$, although valuable for the detection of inflammation and oedema in acute injury [53], typically lacks sensitivity in the detection of chronic myocardial infarction. Additionally, the proposed reconstruction framework has been extended to incorporate nonrigid respiratory motion correction to reconstructed volumes and maps. The proposed framework provides 3D whole‐heart coregistered water and fat volumes and $T_{1}$ and $T_{1\rho }$ maps acquired under free‐breathing at 0.55 T, which offers considerable advantages over conventional 2D breath‐hold mapping sequences typically acquired during a CMR examination.

The proposed technique demonstrated good correlation with 2D SE reference values in phantoms. Generally, vials with large $T_{1}$ or $T_{1\rho }$ were less accurate and precise since the sequence is designed to be accurate and precise for typical myocardial tissue, that is, for $T_{1}$, there are only four HBs between IR pulses, and, for $T_{1\rho }$, the proposed sequence only deploys a single $T_{1\rho }$‐prep with a 40ms duration. The proposed sequence was also compared to in vivo 2D MOLLI and a 2D $T_{1\rho }$ mapping sequence in 11 healthy subjects. Mean $T_{1}$ values measured with the proposed mapping were significantly larger than 2D MOLLI which could be explained by the known underestimation of $T_{1}$ by MOLLI [12]. A similar bias between 3D proposed $T_{1}$ and 2D MOLLI was additionally observed in a phantom experiment (see Figure S3). Mean $T_{1\rho }$ values measured with the proposed mapping were significantly smaller than 2D $T_{1\rho }$. This bias was larger than that observed between 3D proposed $T_{1\rho }$ and 2D $T_{1\rho }$ in a phantom experiment (Figure S3). The large in vivo $T_{1\rho }$ bias observed could be due to physiological confounding factors that are not currently modelled in the dictionary. For example, diffusion effects during gradient spoiling [54] or magnetisation transfer effects [55] that are unaccounted for in the dictionary have been reported to contribute to underestimation of $T_{2}$ in MRF. Although $T_{2}$ underestimation is clearly not the same as $T_{1\rho }$ underestimation, $T_{1\rho }$‐preparation and $T_{2}$‐preparation share several similarities (excitation pulse, refocusing pulses). An underestimation of in vivo $T_{1\rho }$, as well as a discrepancy between phantom and in vivo $T_{1\rho }$, has recently been reported in a 2D cardiac $T_{1}$/$T_{1\rho }$/$T_{2}$ free‐breathing sequence at 3T [56] and in 2D cardiac $T_{1}$/$T_{1\rho }$/$T_{2}$ MRF at 1.5T [48]. We also note that there is no accepted gold standard in vivo 2D cardiac $T_{1\rho }$ mapping sequence at any field strength, and the 2D $T_{1\rho }$ sequence could have a bias in vivo due to physiological factors. If the $T_{1\rho }$ bias decreased with increasing $T_{1\rho }$, this could be problematic as scar tissue (increased $T_{1\rho }$) would be more difficult to distinguish from healthy myocardium, potentially decreasing sensitivity to detect scar. However, phantom experiments indicate the $T_{1\rho }$ bias increases slightly with increasing $T_{1\rho }$, and so we do not expect this bias to particularly decrease sensitivity in patients. Promising results were also obtained when comparing the proposed 3D joint $T_{1}$/$T_{1\rho }$ mapping sequence to 2D LGE in a patient with acute myocarditis, with elevated $T_{1}$ and $T_{1\rho }$ measured in the scar region relative to remote.

There are a number of cardiac MRF sequences that provide an alternative approach to multiparametric mapping than the approach proposed here (see, e.g., [48, 57, 58]). MRF, which uses a pulse sequence of interleaved variable duration magnetisation preparation pulses and variable imaging readout FAs and TRs, has the advantage of allowing more magnetisation preparation modules to provide greater sensitivity to different tissues and can allow estimation of other parameter maps (e.g., $B_{0}$‐/$B_{1}$‐field, proton density) albeit at an exponential increase in computational time. However, MRF acquires many images with highly undersampled k‐space data and is typically performed under breath‐hold and in 2D. The advantages of the proposed approach include generating 3D images of higher image quality than the many lower quality 2D images of MRF, as the k‐space trajectories are much more densely sampled than those used in MRF. Additionally, the proposed approach is performed under free‐breathing, rather than cardiac MRF which is typically acquired under breath‐hold. Also, the proposed approach has four time‐points and is quicker to match with the reconstructed images than MRF which can have a much larger number of points (≈ 100–1000).

Lower SNR is a potential limitation of imaging at 0.55 T. However, we believe that the noise reduction due to the HD‐PROST regularisation deployed in image reconstruction, which has been shown to be robust over different applications and field strengths, for example, coronary magnetic resonance angiography at 1.5T [37] and 3D joint $T_{1}/T_{2}$ mapping at 0.55 T [27], addresses the challenge of lower SNR. We note that the midventricular CV values obtained with the proposed approach here are similar to those obtained with a recently proposed 2D $T_{1}/T_{2}/T_{1\rho }$ MRF sequence at 1.5T [48], 3D proposed vs. 2D MRF CV $T_{1}$ (6.4%±0.9% vs. 6.0%±0.9%) and CV $T_{1\rho }$ (11.1%±1.4% vs. 12.6%±0.8%), which demonstrates comparable precision to multi‐parametric mapping sequences at higher field tstrengths can be achieved with the proposed approach.

This study has some limitations. First, a potential limitation of the sequence is that it could be sensitive to arrhythmias, potentially causing a loss of accuracy and precision in mapping values. A phantom experiment was performed to assess the effect of HR variability during acquisition (Figure S3). A slight increase in $T_{1}$/$T_{1\rho }$ CV with increased HR variability was observed, although the proposed approach did demonstrate some robustness to HR variability. In future work, integration of arrhythmia rejection into the sequence will be considered for subjects with high HR variability. Second, only a single patient was scanned, but further work will include validation of the proposed 3D joint $T_{1}$/$T_{1\rho }$ mapping sequence in a larger cohort of patients with different cardiovascular diseases. Third, the maximal SLA and TSL of $T_{1\rho }$‐preparation are limited both by the maximum power of the RF amplifier as well as SAR constraints. In this study, the SLA and TSL were limited to 150Hz and 40 ms, respectively, due to hardware constraints of the RF power amplifier (RFPA) available in the scanner, but the sequence was well within SAR limits for each subject as expected at 0.55 T since $\text{SAR}\propto |B_{0}{|}^{2}$. The SLA and TSL were chosen conservatively to ensure the same $T_{1\rho }$‐preparation could be run successfully in all subjects, including subjects with larger body mass index (BMI). The low SLA used in this study may limit immediate clinical utility of the sequence, as previous studies at 1.5T have demonstrated increased dispersion in myocardial tissue at higher SLA [59]. Additionally, the relatively low SLA may make the $T_{1\rho }$‐preparation susceptible to $B_{0}$ inhomogeneities ($\Delta B_{0}$). In particular, the offset angle, θ, the tilt of the effective spin‐lock field due to $B_{0}$ inhomogeneities, is defined through $\tan(\theta )=\frac{\Delta B_{0}}{B_{SL}}$ [60]. Thus, smaller $B_{SL}$ or larger $\Delta B_{0}$ increases θ decreasing the efficiency of $T_{1\rho }$‐preparation leading to banding artifacts [61]. The $T_{1\rho }$‐preparation used here has been used with SLA=350 Hz in a previous study at 1.5T [48]. However, $B_{0}$ inhomogeneities at 1.5T are significantly larger than 0.55 T [32], and the relationship between $\Delta B_{0}$ and field‐strength is approximately linear [25]. Thus, in our study at 0.55 T, we may expect that, although the SLA is reduced compared to this previous study, this may be compensated by the reduction in $B_{0}$ field inhomogeneities. We did not observe obvious artifacts due to $B_{0}$ inhomogeneities in the proposed 3D joint $T_{1}$/$T_{1\rho }$ reconstruction in phantom and in vivo experiments. Nevertheless, our preliminary results in a patient indicated that $T_{1\rho }$ at SLA =150 Hz can detect the abnormal myocardium. Overall, more experiments are required to further validate $T_{1\rho }$ mapping at low field.

For further work, firstly, we will consider replacing the continuous‐wave $T_{1\rho }$‐preparation pulse used in this study with adiabatic $T_{1\rho }$‐preparation [62, 63, 64]. With such a modification, and/or improvements in RF amplifier hardware, a significant increase in the achievable maximum SLA may be possible, matching those typically used at higher field strengths. This will better match physical processes of macromolecular–water interactions in infarcted areas of myocardium (i.e., with increased collagen), thus increasing the sensitivity of the proposed mapping to detect fibrosis and further increase robustness to $B_{0}$ inhomogeneity during $T_{1\rho }$‐preparation. Second, we will consider increasing the TSL to ≈50−60 ms and the inclusion of an additional $T_{1\rho }$‐prep module (with a different TSL) to improve sensitivity to $T_{1\rho }$, as well as the inclusion of an additional $T_{2}$‐prep module to explore the possibility of 3D joint $T_{1}$/$T_{1\rho }$/$T_{2}$ mapping. Third, the water‐fat separation component of the sequence was treated qualitatively in vivo here, but future studies will include comprehensive quantitative validation in vivo. Sequences with three or more echoes, to enable correction for $T_{2}^{*}$ relaxation and additionally provide proton density fat‐fraction quantification [58], will also be considered.

## 5 Conclusions

A novel, free‐breathing, 3D joint $T_{1}$/ $T_{1\rho }$ mapping sequence with Dixon encoding was proposed to provide coregistered 3D $T_{1}$ and $T_{1\rho }$ maps and water‐fat volumes with isotropic spatial resolution in a single scan for comprehensive contrast‐agent free myocardial tissue characterization and visualisation of the whole‐heart anatomy on the latest 0.55‐T scanner generation. When comparing to conventional techniques, 3D joint $T_{1}$/$T_{1\rho }$ mapping and fat imaging results demonstrate good quantitative agreement in phantoms and promising results in 11 healthy subjects and 1 patient with acute myocarditis.

## Author Contributions

**Michael G. Crabb:** conceptualization, software (numerical simulations and implementation of research sequence and reconstruction framework), methodology, data acquisition, formal analysis, writing – original draft, writing – review and editing. **Karl P. Kunze:** software (implementation of research sequence and reconstruction framework), writing – review and editing. **Carlos Castillo‐Passi:** data acquisition, writing – review and editing. **Dongyue Si:** data acquisition, writing – review and editing. **Simon J. Littlewood:** data acquisition, writing – review and editing. **Claudia Prieto:** conceptualization, methodology, writing – review and editing, funding acquisition. **René M. Botnar:** conceptualization, methodology, supervision, writing – review and editing, funding acquisition.

## Conflicts of Interest

K.P. Kunze is an employee of Siemens Healthcare Limited. The remaining authors declare no potential conflicts of interest.

## Supporting information




**Figure S1:** Comparison of proposed 3D joint *T*_1_/*T*_1*ρ*_ values to 2D inversion‐recovery (IR) spin‐echo (SE) and 2D *T*_1*ρ*_‐prep SE reference maps for the *T*_1_‐MES and in‐house *T*_1_/*T*_1*ρ*_ phantoms as a function of simulated heart rate (HR) = [60, 80, 100, 120] bpm. Left: Scatter plots including the coefficient of determination from a linear fit, and the black dashed lines are the identity line. Good agreement is observed between 3D proposed and 2D SE references with an *r* ^2^ ≥ 0.976 over all simulated HR and *T*_1_/*T*_1*ρ*_. Right: Bar charts of mean and coefficient of variation (CV) estimation for each vial for proposed 3D joint *T*_1_/*T*_1*ρ*_ mapping.
**Figure S2:** Left: Comparison of fat image of proposed 3D joint *T*_1_/*T*_1*ρ*_ sequence (third volume) to 3D VIBE Dixon reference in an in‐house water‐fat phantom with simulated heart rate (HR) = 60 bpm. Middle: Scatter plot comparison of fat signal of proposed 3D sequence to 3D VIBE Dixon. Good agreement is observed for fat signal between proposed 3D joint *T*_1_/*T*_1*ρ*_ sequence and 3D VIBE Dixon reference with an *r* ^2^ = 0.988. Right: Bland–Altman plot comparison of fat signal of proposed 3D sequence to 3D VIBE Dixon.
**Figure S3:** Proposed 3D joint *T*_1_/*T*_1*ρ*_ mapping in the T1‐MES and in‐house T1/T1ρ phantoms with simulated heart rate (HR) variability. Normally distributed RR intervals were used with mean RR = 1000 ms. Four experiments were performed with increasing RR standard deviation (SD) = 0, 100, 200, 300 ms. Mean/coefficient of variation (CV) *T*_1_/*T*_1*ρ*_ values are plotted as a function of RR SD. Vials with *T*_1_ ≤ 1300 ms and *T*_1*ρ*_ ≤ 150 ms were selected to focus on the typical myocardial tissue value range. Colored lines represent vials with different *T*_1_ or *T*_1*ρ*_ relaxation times. A slight increase in *T*_1_/*T*_1*ρ*_ CV with increased RR SD is observed, but the results demonstrate that the proposed approach does provide some robustness to HR variability.
**Figure S4:** Comparison between proposed 3D joint *T*_1_/*T*_1*ρ*_ mapping, 2D breath‐hold (BH), and 2D spin‐echo (SE) references in the *T*_1_‐MES and in‐house *T*_1_/*T*_1*ρ*_ phantoms with simulated heart rate (HR) = 60 bpm using Bland–Altman plots. Top/bottom: *T*_1_/*T*_1*ρ*_ measurements. Left/middle/right: Proposed 3D joint *T*_1_/*T*_1*ρ*_ versus 2D spin‐echo/2D BH versus 2D SE/proposed 3D joint *T*_1_/*T*_1*ρ*_ versus 2D BH. Vials with *T*_1_ ≤ 1300 ms and *T*_1*ρ*_ ≤ 150 ms were selected to focus on the typical myocardial tissue value range.
**Figure S5:** (a) Proposed 3D *T*_1_, *T*_1*ρ*_, and sum of squared differences (SSD) maps for the *T*_1_‐MES and in‐house *T*_1_/*T*_1*ρ*_ phantoms (simulated HR = 60 bpm). Mean SSD is small for all phantom vials, with mean SSD < 6.1 × 10^−5^ for all vials across the typical myocardial tissue value range (*T*_1_ ≤ 1300 ms and *T*_1*ρ*_ ≤ 150 ms). (b) In vivo proposed 3D *T*_1_, *T*_1*ρ*_, and SSD maps in one representative subject in mid‐ventricular short‐axis (mid‐SAx) view. Mid‐SAx septum and lateral wall *T*_1_, *T*_1*ρ*_, and SSD values are displayed. Left‐ventricle mean SSD for the proposed sequence averaged across all 11 healthy subjects was measured as SSD = (8.89 ± 3.58) × 10^−4^.
**Figure S6:** Violin plots of mean and coefficient of variation (CV) myocardium values for proposed 3D joint *T*_1_/*T*_1*ρ*_ mapping sequence across all healthy subjects and all 16 AHA segments. Ba = Basal, Mi = Mid, Ap = Apex. A = Anterior, I = Inferior, S = Septal, L = Lateral.
**Table S1:** Mean/coefficient of variation (CV) myocardium *T*_1_ and *T*_1*ρ*_ values measured with the proposed 3D whole‐heart joint *T*_1_/*T*_1*ρ*_ mapping sequence averaged across 11 healthy subjects in different regions of the heart.
