# Supplementary material for: 3D Whole‐Heart Joint T1/T1ρ Mapping and Water‐Fat Imaging on a Clinical 0.55‐T Low‐Field Scanner
Source: NMR Biomed. 2025 Dec 22;39(2):e70195. doi: 10.1002/nbm.70195 (PMC12723209; doi:10.1002/nbm.70195)
Supplement: Supplementary file 1 — Figure S1: Comparison of proposed 3D joint T 1/T 1ρ values to 2D inversion‐recovery (IR) spin‐echo (SE) and 2D T 1ρ ‐prep SE reference maps for the T 1‐MES and in‐house T 1/T 1ρ phantoms as a function of simulated heart rate (HR) = [60, 80, 100, 120] bpm. Left: Scatter plots including the coefficient of determination from a linear fit, and the black dashed lines are the identity line. Good agreement is observed between 3D proposed and 2D SE references with an r 2 ≥ 0.976 over all simulated HR and T 1/T 1ρ . Right: Bar charts of mean and coefficient of variation (CV) estimation for each vial for proposed 3D joint T 1/T 1ρ mapping. Figure S2: Left: Comparison of fat image of proposed 3D joint T 1/T 1ρ sequence (third volume) to 3D VIBE Dixon reference in an in‐house water‐fat phantom with simulated heart rate (HR) = 60 bpm. Middle: Scatter plot comparison of fat signal of proposed 3D sequence to 3D VIBE Dixon. Good agreement is observed for fat signal between proposed 3D joint T 1/T 1ρ sequence and 3D VIBE Dixon reference with an r 2 = 0.988. Right: Bland–Altman plot comparison of fat signal of proposed 3D sequence to 3D VIBE Dixon. Figure S3: Proposed 3D joint T 1/T 1ρ mapping in the T1‐MES and in‐house T1/T1ρ phantoms with simulated heart rate (HR) variability. Normally distributed RR intervals were used with mean RR = 1000 ms. Four experiments were performed with increasing RR standard deviation (SD) = 0, 100, 200, 300 ms. Mean/coefficient of variation (CV) T 1/T 1ρ values are plotted as a function of RR SD. Vials with T 1 ≤ 1300 ms and T 1ρ ≤ 150 ms were selected to focus on the typical myocardial tissue value range. Colored lines represent vials with different T 1 or T 1ρ relaxation times. A slight increase in T 1/T 1ρ CV with increased RR SD is observed, but the results demonstrate that the proposed approach does provide some robustness to HR variability. Figure S4: Comparison between proposed 3D joint T 1/T 1ρ mapping, 2D breath‐hold (BH), and 2D spin‐echo (SE) [file NBM-39-e70195-s001.pdf]

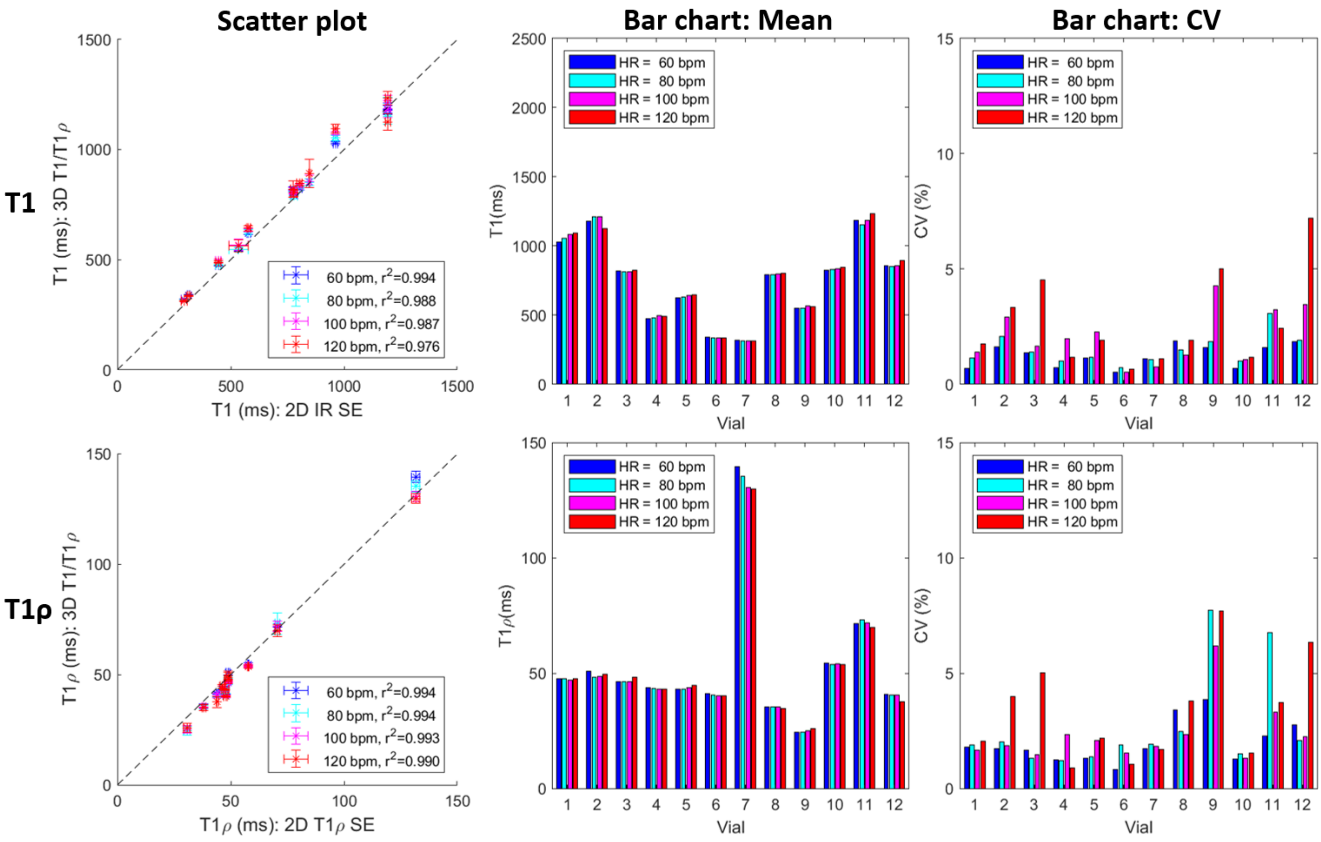

Figure S1: Comparison of proposed 3D joint $T_1/T_{1\rho}$ values to 2D inversion-recovery (IR) spin-echo (SE) and 2D $T_{1\rho}$-prep SE reference maps for the T_1-MES and in-house $T_1/T_{1\rho}$ phantoms as a function of simulated heart-rate (HR) $= [60, 80, 100, 120]$ bpm. Left: Scatter plots including the coefficient of determination from a linear fit, and the black dashed lines are the identity line. Good agreement is observed between 3D proposed and 2D SE references with an $r^2 \geq 0.976$ over all simulated HR and $T_1/T_{1\rho}$. Right: Bar charts of mean and coefficient of variation (CV) estimation for each vial for proposed 3D joint $T_1/T_{1\rho}$ mapping.

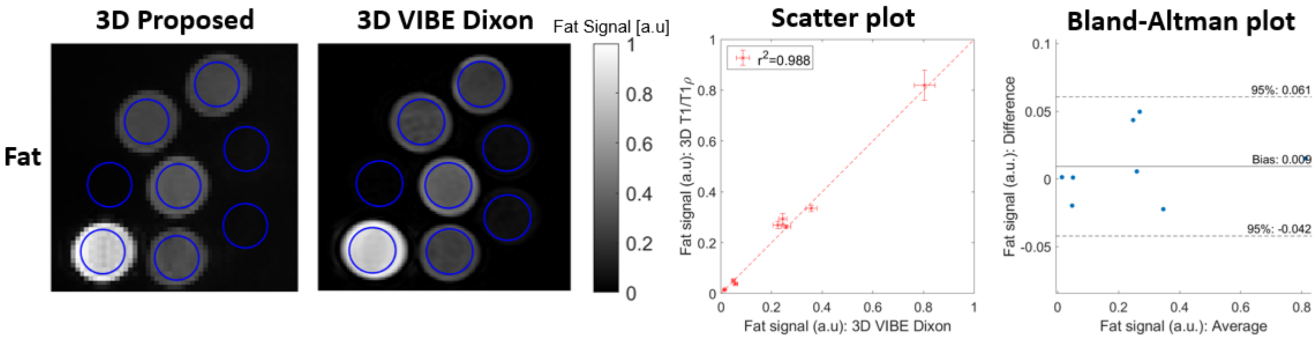

Figure S2: Left: Comparison of fat image of proposed 3D joint $T_1/T_{1\rho}$ sequence (3rd volume) to 3D VIBE Dixon reference in an in-house water-fat phantom with simulated heart-rate (HR) $= 60$ bpm. Middle: Scatter plot comparison of fat signal of proposed 3D sequence to 3D VIBE Dixon. Good agreement is observed for fat signal between proposed 3D joint $T_1/T_{1\rho}$ sequence and 3D VIBE Dixon reference with an $r^2 = 0.988$. Right: Bland-Altman plot comparison of fat signal of proposed 3D sequence to 3D VIBE Dixon.

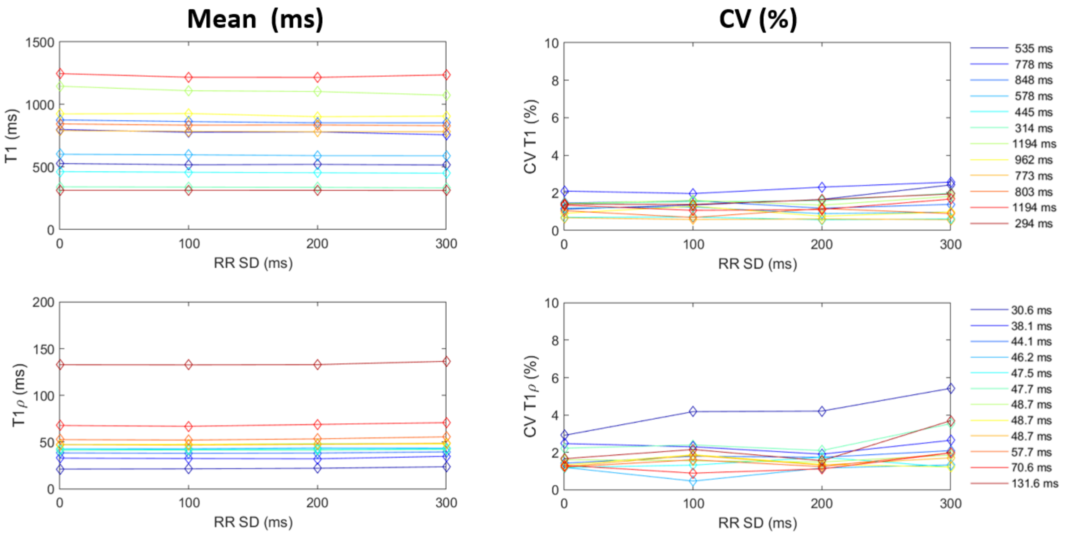

Figure S3: Proposed 3D joint $T_1/T_{1\rho}$ mapping in the T_1-MES and in-house $T_1/T_{1\rho}$ phantoms with simulated heart-rate (HR) variability. Normally distributed RR intervals were used with mean RR = 1000 ms. Four experiments were performed with increasing RR standard deviation (SD)= $0, 100, 200, 300$ ms. Mean/coefficient of variation (CV) $T_1/T_{1\rho}$ values are plotted as a function of RR SD. Vials with $T_1 \leq 1300\,\text{ms}$ and $T_{1\rho} \leq 150\,\text{ms}$ were selected to focus on the typical myocardial tissue value range. Coloured lines represent vials with different T_1 or $T_{1\rho}$ relaxation times. A slight increase in $T_1/T_{1\rho}$ CV with increased RR SD is observed, but the results demonstrate that the proposed approach does provide some robustness to HR variability.

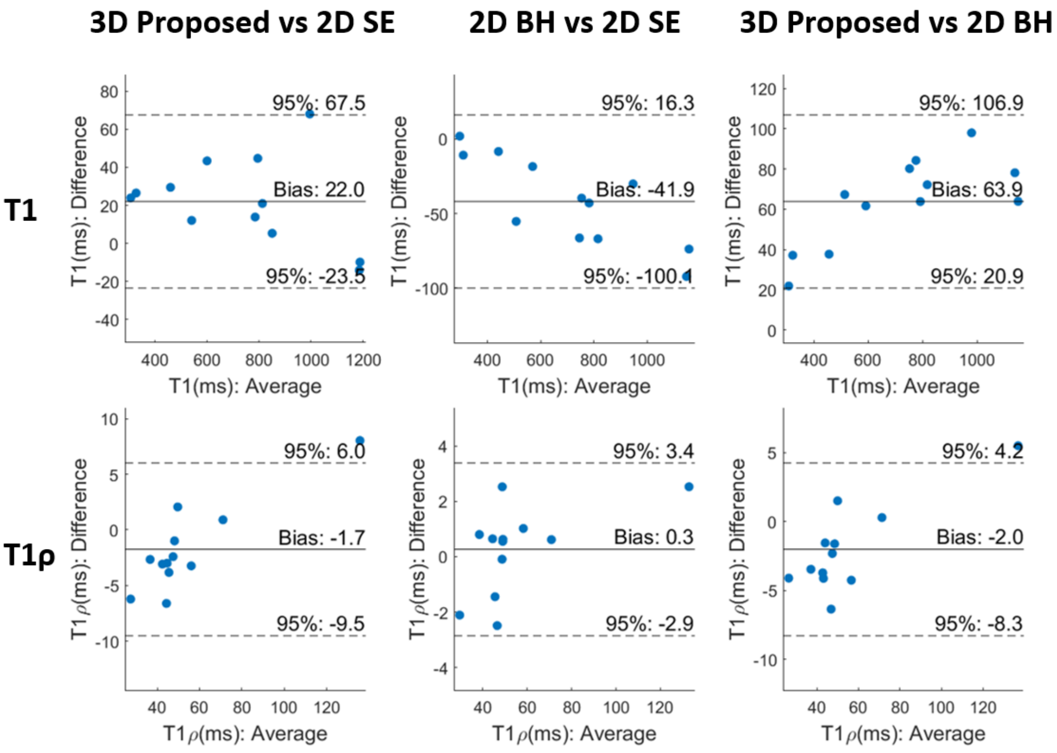

Figure S4: Comparison between proposed 3D joint $T_1/T_{1\rho}$ mapping, 2D breath-hold (BH) and 2D spin-echo (SE) references in the T_1-MES and in-house $T_1/T_{1\rho}$ phantoms with simulated heart-rate (HR)=60 bpm using Bland-Altman plots. Top/Bottom: $T_1/T_{1\rho}$ measurements. Left/Middle/Right: Proposed 3D joint $T_1/T_{1\rho}$ vs 2D Spin-Echo/2D BH vs 2D SE/proposed 3D joint $T_1/T_{1\rho}$ vs 2D BH. Vials with $T_1 \leq 1300\,\text{ms}$ and $T_{1\rho} \leq 150\,\text{ms}$ were selected to focus on the typical myocardial tissue value range.

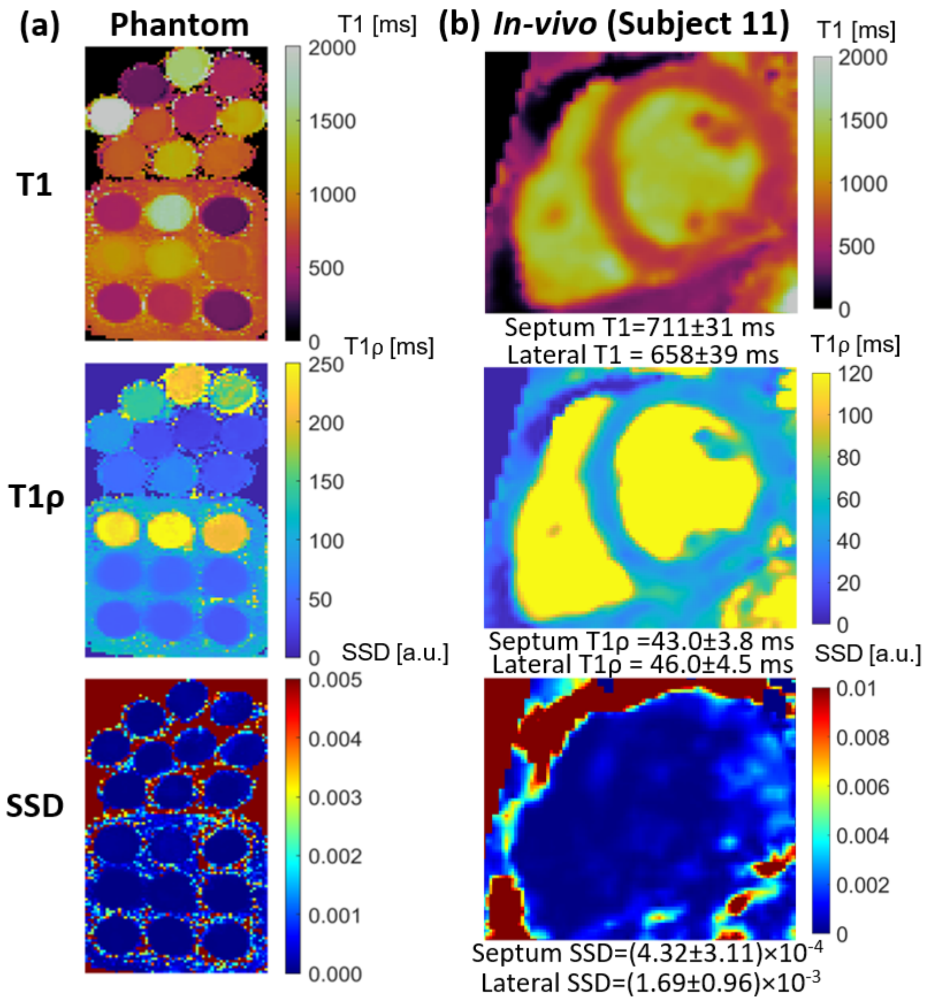

Figure S5: (a) Proposed 3D T_1, $T_{1\rho}$ and sum of squared differences (SSD) maps for the T_1-MES and in-house $T_1/T_{1\rho}$ phantoms (simulated HR $= 60\,$bpm). Mean SSD is small for all phantom vials, with mean SSD $< 6.1 \times 10^{-5}$ for all vials across the typical myocardial tissue value range ($T_1 \leq 1300\,$ms and $T_{1\rho} \leq 150\,$ms). (b) *In-vivo* proposed 3D T_1, $T_{1\rho}$ and SSD maps in 1 representative subject in mid-ventricular short-axis (mid-SAx) view. Mid-SAx septum and lateral wall T_1, $T_{1\rho}$ and SSD values are displayed. Left-ventricle mean SSD for the proposed sequence averaged across all 11 healthy subjects was measured as SSD $= (8.89 \pm 3.58) \times 10^{-4}$.

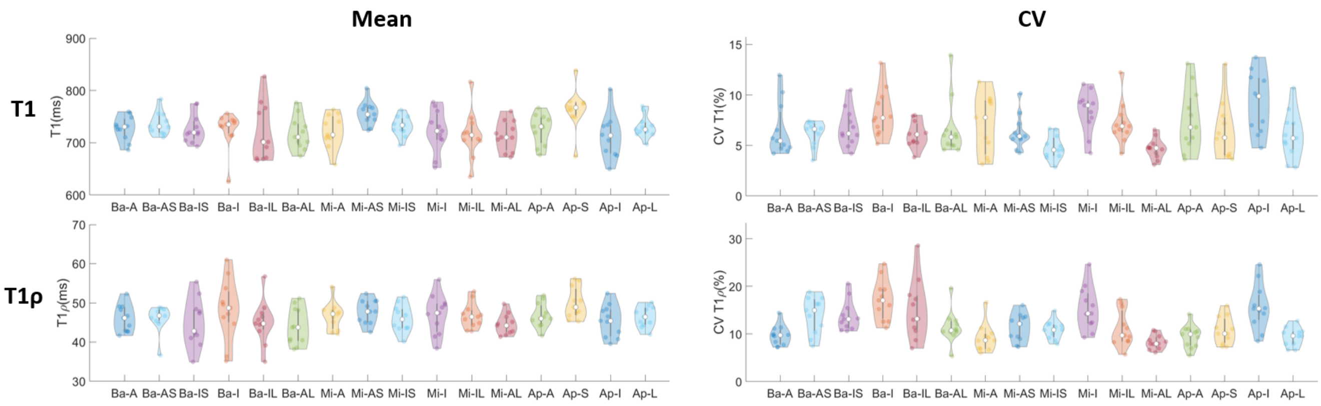

Figure S6: Violin plots of mean and coefficient of variation (CV) myocardium values for proposed 3D joint $T_1/T_{1\rho}$ mapping sequence across all healthy subjects and all 16 AHA segments. Ba=Basal, Mi=Mid, Ap=Apex. A=Anterior, I=Inferior, S=Septal, L=Lateral.

	Mean (ms)		CV (%)	
	**T_1**	**$T_{1\rho}$**	**T_1**	**$T_{1\rho}$**
**Mid-Septal**	744 ± 19	46.9 ± 2.7	6.0 ± 1.0	12.0 ± 2.0
**Apex**	733 ± 24	47.1 ± 2.6	7.2 ± 1.5	11.2 ± 1.8
**Mid**	727 ± 23	46.5 ± 2.3	6.4 ± 0.9	11.1 ± 1.4
**Base**	725 ± 19	45.7 ± 2.8	6.7 ± 1.2	13.6 ± 2.5
**Anterior**	725 ± 24	46.4 ± 3.0	7.0 ± 1.4	9.4 ± 1.3
**Inferior**	720 ± 33	46.9 ± 4.8	8.6 ± 1.9	16.2 ± 3.2
**Septal**	743 ± 19	46.9 ± 2.7	6.1 ± 0.8	12.1 ± 1.6
**Lateral**	719 ± 29	45.4 ± 2.7	6.1 ± 1.0	11.1 ± 1.8
**Left-Ventricle**	728 ± 20	46.3 ± 2.3	6.7 ± 0.7	12.1 ± 1.2

Table S1: Mean/coefficient of variation (CV) myocardium T_1 and $T_{1\rho}$ values measured with the proposed 3D whole-heart joint $T_1/T_{1\rho}$ mapping sequence averaged across 11 healthy subjects in different regions of the heart.
